# Information Theoretic Security for Shannon Cipher System under Side-Channel Attacks †

**DOI:** 10.3390/e21050469

**Published:** 2019-05-05

**Authors:** Bagus Santoso, Yasutada Oohama

**Affiliations:** University of Electro-Communications, 1-5-1 Chofugaoka, Tokyo 182-8585, Japan

**Keywords:** information theoretic security, side-channel attacks, Shannon cipher system, one helper source coding problem, strong converse theorem

## Abstract

In this paper, we propose a new theoretical security model for Shannon cipher systems under side-channel attacks, where the adversary is not only allowed to collect ciphertexts by eavesdropping the public communication channel but is also allowed to collect the physical information leaked by the devices where the cipher system is implemented on, such as running time, power consumption, electromagnetic radiation, etc. Our model is very robust as it does not depend on the kind of physical information leaked by the devices. We also prove that in the case of one-time pad encryption, we can strengthen the secrecy/security of the cipher system by using an appropriate affine encoder. More precisely, we prove that for any distribution of the secret keys and any measurement device used for collecting the physical information, we can derive an achievable rate region for reliability and security such that if we compress the ciphertext using an affine encoder with a rate within the achievable rate region, then: (1) anyone with a secret key will be able to decrypt and decode the ciphertext correctly, but (2) any adversary who obtains the ciphertext and also the side physical information will not be able to obtain any information about the hidden source as long as the leaked physical information is encoded with a rate within the rate region. We derive our result by adapting the framework of the one helper source coding problem posed and investigated by Ahlswede and Körner (1975) and Wyner (1975). For reliability and security, we obtain our result by combining the result of Csizár (1982) on universal coding for a single source using linear codes and the exponential strong converse theorem of Oohama (2015) for the one helper source coding problem.

## 1. Introduction

In most of theoretical security models for encryption schemes, the adversary only obtains information from the public communication channel. In such models, an adversary is often treated as an entity that tries to obtain information about the hidden source only from the ciphertexts that are sent through the public communication channel. However, in the real world, the encryption schemes are implemented on physical electronic devices, and it is widely known that any process executed in an electronic circuit will generate a certain kind of correlated physical phenomena as “side” effects, according to the type of process. For example, differences in inputs to a process in an electronic circuit can induce differences in the heat, power consumption, and electromagnetic radiation generated as byproducts by the devices. Therefore, we may consider that an adversary who has a certain degree of physical access to the devices may obtain some information on very sensitive hidden data, such as the keys used for the encryption, just by measuring the generated physical phenomena using appropriate measurement devices. More precisely, an adversary may deduce the value of the bits of the key by measuring the differences in the timing of the process of encryption or the differences in the power consumption, electromagnetic radiation, and other physical phenomena. This information channel where the adversary obtains data in the form of physical phenomena is called the *side-channel*, and attacks using the side-channel are known as side-channel attacks.

In the literature, there have been many works showing that adversaries have succeeded in breaking the security of cryptographic systems by exploiting side-channel information such as running time, power consumption, and electromagnetic radiation in the real physical world [1,2,3,4,5].

### 1.1. Our Contributions

#### 1.1.1. Security Model for Side-Channel Attacks

**Figure 1:**
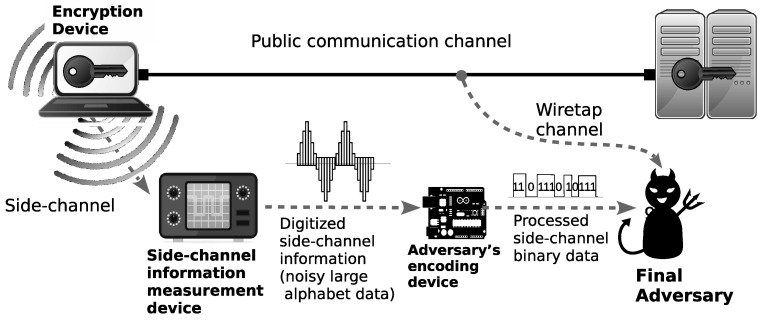
Illustration of side-channel attacks.

In this paper, we propose a security model where the adversary attempts to obtain information about the hidden source by collecting data from (1) the public communication channel in the form of ciphertexts, and (2) the side-channel in the form of some physical data related to the encryption keys. Our proposed security model is illustrated in Figure 1.

Based on the security model illustrated above, we formulate a security problem of strengthening the security of Shannon cipher system where the encryption is implemented on a physical encryption device and the adversary attempts to obtain some information on the hidden source by collecting ciphertexts and performing side-channel attacks.

We describe our security model in a more formal way as follows. The source *X* is encrypted using an encryption device with secret key *K* installed. The result of the encryption, i.e., ciphertext *C*, is sent through a public communication channel to a data center where *C* is decrypted back into the source *X* using the same key *K*. The adversary A is allowed to obtain *C* from the public communication channel and is also equipped with an encoding device $\varphi_{A}$ that encodes and processes the noisy large alphabet data *Z*, i.e., the measurement result of the physical information obtained from the side-channel, into the appropriate binary data $M_{A}$. It should be noted that in our model, we do not put any limitation on the kind of physical information measured by the adversary. Hence, any theoretical result based on this model automatically applies to any kind of side-channel attack, including timing analysis, power analysis, and electromagnetic (EM) analysis. In addition, the measurement device may just be a simple analog-to-digital converter that converts the analog data representing physical information leaked from the device into “noisy” digital data *Z*. In our model, we represent the measurement process as a communication channel *W*.

#### 1.1.2. Main Result

As the main theoretical result, we show that we can strengthen the secrecy/security of the Shannon cipher implemented on a physical device against an adversary who collects the ciphertexts and launches side-channel attacks by a simple method of compressing the ciphertext *C* from a Shannon cipher using an affine encoder φ into $\tilde{C}$ before releasing it into the public communication channel.

We prove that in the case of one-time pad encryption, we can strengthen the secrecy/security of the cipher system by using an appropriate affine encoder. More precisely, we prove that for any distribution of the secret key *K* and any measurement device (used to convert the physical information from a side-channel into the noisy large alphabet data *Z*), we can derive an achievable rate region for $\left(R_{A},R\right)$ such that if we compress the ciphertext *C* into $\tilde{C}$ using the affine encoder φ, which has an encoding rate *R* inside the achievable region, then we can achieve reliability and security in the following sense:

- anyone with secret key *K* can construct an appropriate decoder that decrypts and encodes $\tilde{C}$ with exponentially decaying error probability, but
- the amount of information gained by any adversary A who obtains the compressed ciphertext $\tilde{C}$ and encoded physical information $M_{A}$ is exponentially decaying to zero as long as the encoding device $\varphi_{A}$ encodes the side physical information into $M_{A}$ with a rate $R_{A}$ within the achievable rate region.

By utilizing the homomorphic property of one-time-pad and affine encoding, we are able to separate the theoretical analysis of reliability and security such that we can deal with each issue independently. For reliability, we mainly obtain our result by using the result of Csizár [6] on the universal coding for a single source using linear codes. For the security analysis, we derive our result by adapting the framework of the one helper source coding problem posed and investigated by Ahlswede, Körner [7] and Wyner [8]. Specifically, in order to derive the secrecy exponent, we utilize the exponential strong converse theorem of Oohama [9] for the one helper source coding problem. In [10], Watanabe and Oohama deal with a similar source coding problem, but their result is insufficient for deriving the lower bound of the secrecy exponent. We will explain the relation between our method and previous related works in more detail in Section 4.

### 1.2. Comparison to Existing Models of Side-Channel Attacks

The most important feature of our model is that we do not make any assumption about the type or characteristics of the physical information that is measured by the adversary. Several theoretical models analyzing the security of a cryptographic system against side-channel attacks have been proposed in the literature. However, most of the existing works are applicable only for specific characteristics of the leaked physical information. For example, Brier et al. [1] and Coron et al. [11] propose a statistical model for side-channel attacks using the information from power consumption and the running time, whereas Agrawal et al. [5] propose a statistical model for side-channel attacks using electromagnetic (EM) radiations. A more general model for side-channel attacks is proposed by Köpf et al. [12] and Backes et al. [13], but they are heavily dependent upon implementation on certain specific devices. Micali et al. [14] propose a very general security model to capture the side-channel attacks, but they fail to offer any hint of how to build a concrete countermeasure against the side-channel attacks. The closest existing model to ours is the general framework for analyzing side-channel attacks proposed by Standaert et al. [15]. The authors of [15] propose a countermeasure against side-channel attacks that is different from ours, i.e., noise insertion on implementation. It should be noted that the noise insertion countermeasure proposed by [15] is dependent on the characteristics of the leaked physical information. On the other hand, our countermeasure, i.e., compression using an affine encoder, is independent of the characteristics of the leaked physical information.

### 1.3. Comparison to Encoding before Encryption

In this paper, our proposed solution is to perform additional encoding in the form of compression after the encryption process. Our aim is that by compressing the ciphertext, we compress the key “indirectly” and increase the “flatness” of the key used in the compressed ciphertext ($\tilde{C}$) such that the adversary will not get much additional information from eavesdropping on the compressed ciphertext ($\tilde{C}$). Instead of performing the encoding after encryption, one may consider performing the encoding before encryption, i.e., encoding the source and the key “directly” before performing the encryption. However, since we need to apply two separate encodings on the source and the key, we can expect that the implementation cost is more expensive than our proposed solution, i.e., approximately double the cost of applying our proposed solution. Moreover, it is not completely clear whether our security analysis still applies for this case. For example, if the adversary performs the side-channel attacks on the key after it is encoded (before encryption), we need a complete remodeling of the security problem.

### 1.4. Organization of this Paper

This paper is structured as follows. In Section 2, we show the basic notations and definitions that we use throughout this paper, and we also describe the formal formulations of our model and the security problem. In Section 3, we explain the idea and the formulation of our proposed solution. In Section 4, we explain the relation between our formulation and previous related works. Based on this, we explain the theoretical challenge which we have to overcome to prove that our proposed solution is sound. In Section 5, we state our main theorem on the reliability and security of our solution. In Section 6, we show the proof of our main theorem. We put the proofs of other related propositions, lemmas, and theorems in the appendix.

## 2. Problem Formulation

In this section, we will introduce the general notations used throughout this paper and provide a description of the basic problem we are focusing on, i.e., side-channel attacks on Shannon cipher systems. We also explain the basic framework of the solution that we consider to solve the problem. Finally, we state the formulation of the reliability and security problem that we consider and aim to solve in this paper.

### 2.1. Preliminaries

In this subsection, we show the basic notations and related consensus used in this paper.

*Random Source of Information and Key:* Let *X* be a random variable from a finite set X. Let ${\left\{X_{t}\right\}}_{t=1}^{\infty }$ be a stationary discrete memoryless source (DMS) such that for each t=1,2,…, $X_{t}$ takes values in the finite set X and obeys the same distribution as that of *X* denoted by $p_{X}={\left\{p_{X}\left(x\right)\right\}}_{x\in X}$. The stationary DMS ${\left\{X_{t}\right\}}_{t=1}^{\infty }$ is specified with $p_{X}$. In addition, let *K* be a random variable taken from the same finite set X and representing the key used for encryption. Similarly, let ${\left\{K_{t}\right\}}_{t=1}^{\infty }$ be a stationary discrete memoryless source such that for each t=1,2,…, $K_{t}$ takes values in the finite set X and obeys the same distribution as that of *K* denoted by $p_{K}={\left\{p_{K}\left(k\right)\right\}}_{k\in X}$. The stationary DMS ${\left\{K_{t}\right\}}_{t=1}^{\infty }$ is specified with $p_{K}$. In this paper, we assume that $p_{K}$ is the uniform distribution over X. 

*Random Variables and Sequences:* We write the sequence of random variables with length *n* from the information source as follows: $X^{n}:=X_{1}X_{2}\cdots X_{n}$. Similarly, strings with length *n* of $X^{n}$ are written as $x^{n}:=x_{1}x_{2}\cdots x_{n}\in X^{n}$. For $x^{n}\in X^{n}$, $p_{X^{n}}\left(x^{n}\right)$ stands for the probability of the occurrence of $x^{n}$. When the information source is memoryless, specified with $p_{X}$, the following equation holds:

$$p_{X^{n}}\left(x^{n}\right)=\prod_{t=1}^{n}p_{X}\left(x_{t}\right).$$

In this case, we write $p_{X^{n}}\left(x^{n}\right)$ as $p_{X}^{n}\left(x^{n}\right)$. Similar notations are used for other random variables and sequences. 

*Consensus and Notations:* Without loss of generality, throughout this paper, we assume that X is a finite field. The notation ⊕ is used to denote the field addition operation, while the notation ⊖ is used to denote the field subtraction operation, i.e., a⊖b=a⊕(−b), for any elements a,b∈X. Throughout this paper, all logarithms are taken to the natural basis.

### 2.2. Basic System Description

In this subsection, we explain the basic system setting and the basic adversarial model we consider in this paper. First, let the information source and the key be generated independently by different parties $S_{\mathrm{gen}}$ and $K_{\mathrm{gen}}$, respectively. In our setting, we assume the following:

- The random key $K^{n}$ is generated by $K_{\mathrm{gen}}$ from a uniform distribution.
- The source is generated by $S_{\mathrm{gen}}$ and is independent of the key.

**Figure 2:**
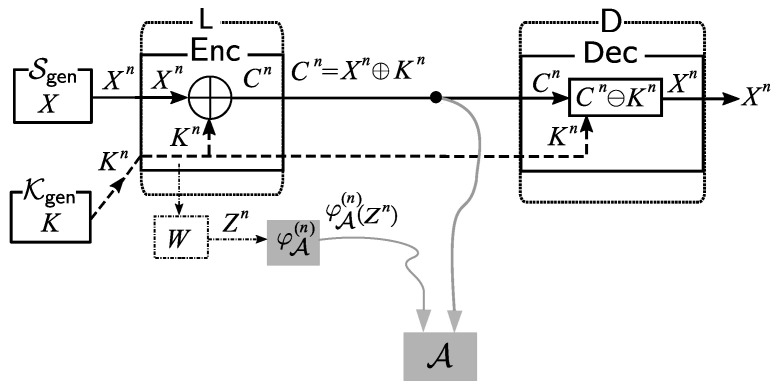
Main problem: side-channel attacks on a Shannon cipher system.

Next, let the random source $X^{n}$ from $S_{\mathrm{gen}}$ be sent to the node L, and let the random key $K^{n}$ from $K_{\mathrm{gen}}$ also be sent to L. Further settings of our system are described as follows and are also shown in Figure 2.

1. *Source Processing:* At the node L, $X^{n}$ is encrypted with the key $K^{n}$ using the encryption function Enc. The ciphertext $C^{n}$ of $X^{n}$ is given by
  $$C^{n}:=\mathrm{Enc}\left(X^{n}\right)=X^{n}⊕K^{n}.$$
2. *Transmission:* Next, the ciphertext $C^{n}$ is sent to the information processing center D through a public communication channel. Meanwhile, the key $K^{n}$ is sent to D through a private communication channel.
3. *Sink Node Processing:* In D, we decrypt the ciphertext $C^{n}$ using the key $K^{n}$ through the corresponding decryption procedure Dec defined by $\mathrm{Dec}\left(C^{n}\right)=C^{n}⊖K^{n}$. It is obvious that we can correctly reproduce the source output $X^{n}$ from $C^{n}$ and $K^{n}$ with the decryption function Dec.

*Side-Channel Attacks by Eavesdropper Adversary:* An (eavesdropper) adversary A eavesdrops on the public communication channel in the system. The adversary A also uses side information obtained by side-channel attacks. In this paper, we introduce a new theoretical model of side-channel attacks that is described as follows. Let Z be a finite set and let W:X→Z be a noisy channel. Let *Z* be a channel output from *W* for the random input variable *K*. We consider the discrete memoryless channel specified with *W*. Let $Z^{n}\in Z^{n}$ be a random variable obtained as the channel output by connecting $K^{n}\in X^{n}$ to the input channel. We write a conditional distribution on $Z^{n}$ given $K^{n}$ as

$$W^{n}={\left\{W^{n}\left(z^{n}|k^{n}\right)\right\}}_{\left(k^{n},z^{n}\right)\in K^{n}\times Z^{n}}.$$

Since the channel is memoryless, we have

(1) $$W^{n}\left(z^{n}|k^{n}\right)=\prod_{t=1}^{n}W\left(z_{t}|k_{t}\right).$$

On the above output $Z^{n}$ of $W^{n}$ for the input $K^{n}$, we assume the following:

- The three random variables *X*, *K*, and *Z* satisfy X⊥(K,Z), which implies that $X^{n}⊥\left(K^{n},Z^{n}\right)$.
- *W* is given in the system and the adversary A cannot control *W*.
- Through side-channel attacks, the adversary A can access $Z^{n}$.

We next formulate the side information the adversary A obtains by side-channel attacks. For each n=1,2,⋯, let $\varphi_{A}^{\left(n\right)}:Z^{n}\to M_{A}^{\left(n\right)}$ be an encoder function. Set $\varphi_{A}:={\left\{\varphi_{A}^{\left(n\right)}\right\}}_{n=1,2,\cdots }.$ Let

$$R_{A}^{\left(n\right)}:=\frac{1}{n}\log||\varphi_{A}\left|\right|=\frac{1}{n}\log\left|M_{A}^{\left(n\right)}\right|$$

be a rate of the encoder function $\varphi_{A}^{\left(n\right)}$. For $R_{A}>0$, we set

$$F_{A}^{\left(n\right)}\left(R_{A}\right):=\left\{\varphi_{A}^{\left(n\right)}:R_{A}^{\left(n\right)}\leq R_{A}\right\}.$$

For the encoded side information the adversary A obtains, we assume the following.

- The adversary A, having accessed $Z^{n}$, obtains the encoded additional information $\varphi_{A}^{\left(n\right)}\left(Z^{n}\right)$. For each n=1,2,⋯, the adversary A can design $\varphi_{A}^{\left(n\right)}$.
- The sequence ${\left\{R_{A}^{\left(n\right)}\right\}}_{n=1}^{\infty }$ must be upper-bounded by a prescribed value. In other words, the adversary A must use $\varphi_{A}^{\left(n\right)}$ such that for some $R_{A}$ and for any sufficiently large *n*, $\varphi_{A}^{\left(n\right)}\in F_{A}^{\left(n\right)}\left(R_{A}\right)$.

*On the Scope of Our Theoretical Model:* When the |Z| is not so large, the adversary A may directly access $Z^{n}$. In contrast, in a real situation of side-channel attacks, often the noisy version $Z^{n}$ of $K^{n}$ can be regarded as very close to an analog random signal. In this case, |Z| is sufficiently large and the adversary A cannot obtain $Z^{n}$ in a lossless form. Our theoretical model can address such situations of side-channel attacks.

### 2.3. Solution Framework

**Figure 3:**
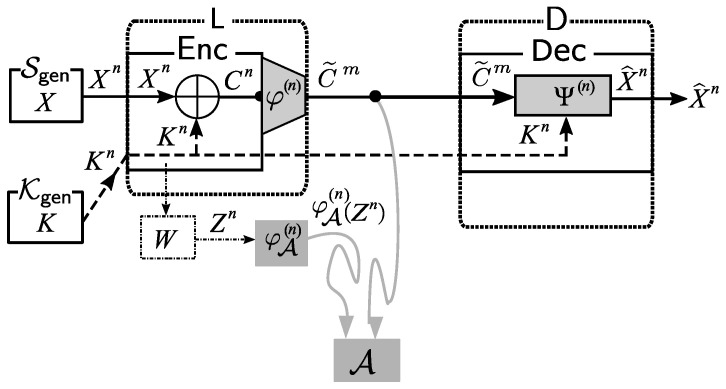
Basic solution framework: post-encryption-compression coding system.

As the basic solution framework, we consider applying a post-encryption-compression coding system. The application of this system is illustrated in Figure 3.

1. *Encoding at Source node*L: We first use $\varphi^{\left(n\right)}$ to encode the ciphertext $C^{n}=X^{n}⊕K^{n}$. The formal definition of $\varphi^{\left(n\right)}$ is $\varphi_{i}^{\left(n\right)}:$$X^{n}\to$$X^{m}$. Let ${\tilde{C}}^{m}=\varphi^{\left(n\right)}\left(C^{n}\right)$. Instead of sending $C^{n}$, we send ${\tilde{C}}^{m}$ to the public communication channel.
2. *Decoding at Sink Nodes*D: D receives ${\tilde{C}}^{m}$ from the public communication channel. Using the common key $K^{n}$ and the decoder function $\Psi^{\left(n\right)}:X^{m}\times X^{n}\to X^{n}$, D outputs an estimation ${\hat{X}}^{n}=\Psi^{\left(n\right)}\left({\tilde{C}}^{m},K^{n}\right)$ of $X^{n}$.

*On Reliability and Security:* From the description of our system in the previous section, the decoding process in our system above is successful if ${\hat{X}}^{n}=X^{n}$ holds. Combining this and (6), it is clear that the decoding error probabilities $p_{e}$ are as follows:

$$\begin{array}{cc} p_{e}= & p_{e}\left(\varphi^{\left(n\right)},\Psi^{\left(n\right)}|p_{X}^{n}\right):=\mathrm{Pr}\left[\Psi^{\left(n\right)}\left(\varphi^{\left(n\right)}\left(X^{n}\right)\right)\neq X^{n}\right]. \end{array}$$

Set $M_{A}^{\left(n\right)}=\varphi_{A}^{\left(n\right)}\left(Z^{n}\right)$. The information leakage $\Delta^{\left(n\right)}$ on $X^{n}$ from $\left({\tilde{C}}^{m},M_{A}^{\left(n\right)}\right)$ is measured by the mutual information between $X^{n}$ and $({\tilde{C}}^{m},$$M_{A}^{\left(n\right)})$. This quantity is formally defined by

$$\begin{array}{c} \Delta^{\left(n\right)}=\Delta^{\left(n\right)}\left(\varphi^{\left(n\right)},\varphi_{A}^{\left(n\right)}|p_{X}^{n},p_{K}^{n},W^{n}\right):=I\left(X^{n};{\tilde{C}}^{m},M_{A}^{\left(n\right)}\right). \end{array}$$

*Reliable and Secure Framework:*

**Definition** **1.** *A quantity R is achievable under*$R_{A}$>0*for the system*Sys*if there exists a sequence*$\{(\varphi^{\left(n\right)},$$\Psi^{\left(n\right)}{)\}}_{n\geq 1}$*such that*∀ϵ>0, $\exists n_{0}=n_{0}\left(\epsilon \right)\in N_{0}$, $\forall n\geq n_{0}$, *we have*

$$\begin{array}{c} \frac{1}{n}\log|X^{m}|=\frac{m}{n}\log\left|X\right|\leq R,p_{e}\left(\varphi^{\left(n\right)},\Psi^{\left(n\right)}|p_{X}^{n}\right)\leq \epsilon , \end{array}$$

*and for any eavesdropper*A*with*$\varphi_{A}$*satisfying*$\varphi_{A}^{\left(n\right)}\in F_{A}^{\left(n\right)}\left(R_{A}\right)$,

$$\begin{array}{c} \Delta^{\left(n\right)}\left(\varphi^{\left(n\right)},\varphi_{A}^{\left(n\right)}|p_{X}^{n},p_{K}^{n},W^{n}\right)\leq \epsilon . \end{array}$$

**Definition** **2.** *[Reliable and Secure Rate Region] Let*$R_{\mathrm{Sys}}(p_{X},$$p_{K},W)$*denote the set of all*$\left(R_{A},R\right)$*such that R is achievable under*$R_{A}$. *We call*$R_{\mathrm{Sys}}(p_{X},p_{K},$W)*the reliable and secure rate region*.

**Definition** **3.** *A triple*(R,E,F)*is achievable under*$R_{A}>0$*for the system*Sys*if there exists a sequence*$\{(\varphi^{\left(n\right)},$$\psi^{\left(n\right)}{)\}}_{n\geq 1}$*such that*∀ϵ>0, $\exists n_{0}=n_{0}\left(\epsilon \right)\in N_{0}$, ∀n$\geq n_{0}$, *we have*

$$\begin{array}{c} \frac{1}{n}\log|X^{m}|=\frac{m}{n}\log\left|X\right|\leq R,p_{e}\left(\phi^{\left(n\right)},\psi^{\left(n\right)}|p_{X}^{n}\right)\leq e^{-n(E-\epsilon )}, \end{array}$$

*and for any eavesdropper*A*with*$\varphi_{A}$*satisfying*$\varphi_{A}^{\left(n\right)}\in F_{A}^{\left(n\right)}\left(R_{A}\right)$, *we have*

$$\begin{array}{c} \Delta^{\left(n\right)}\left(\varphi^{\left(n\right)},\varphi_{A}^{\left(n\right)}|p_{X}^{n},p_{K}^{n},W^{n}\right)\leq e^{-n(F-\epsilon )}. \end{array}$$

**Definition** **4** (Rate, Reliability, and Security Region)**.** *Let*$D_{\mathrm{Sys}}(p_{X},$$p_{K},W)$*denote the set of all*$\left(R_{A},R,E,F\right)$*such that*(R,E,F)*is achievable under*$R_{A}$. *We call*$D_{\mathrm{Sys}}(p_{X},$$p_{K},W)$*the rate, reliability and security region*.

Our aim in this paper is to find the explicit inner bounds of $R_{\mathrm{Sys}}(p_{X},$ $p_{K},W)$ and $D_{\mathrm{Sys}}(p_{X},$ $p_{K},W)$.

## 3. Proposed Idea: Affine Encoder as a Privacy Amplifier

**Figure 4:**
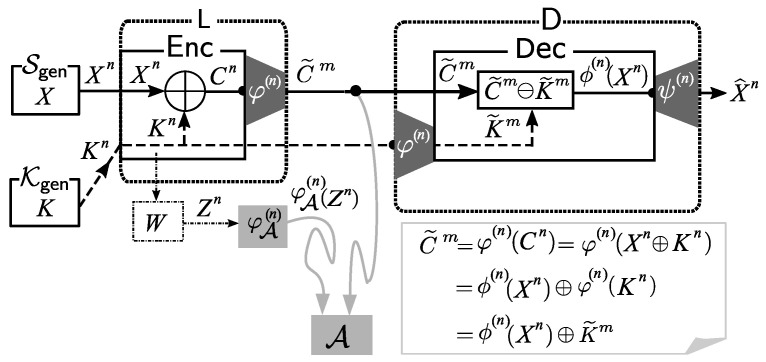
Our proposed solution: affine encoders as privacy amplifiers.

In order to instantiate the basic solution framework mentioned in previous section, we propose the use of an affine encoder as the compression function $\varphi^{\left(n\right)}$. We show in this section that we can easily construct an affine encoder that is suitable for our solution framework based on a linear encoder. The instantiation of the solution framework with an affine encoder is illustrated in Figure 4.

*Construction of the Affine Encoder:* For each n=1,2,⋯, let $\phi^{\left(n\right)}:X^{n}\to X^{m}$ be a linear mapping. We define the mapping $\phi^{\left(n\right)}$ by

(2) $$\phi^{\left(n\right)}\left(x^{n}\right)=x^{n}A\;\mathrm{for}\;x^{n}\in X^{n},$$

where *A* is a matrix with *n* rows and *m* columns. Entries of *A* are from X. We fix $b^{m}\in X^{m}$. Define the mapping $\varphi^{\left(n\right)}:X^{n}\to X^{m}$ by

(3) $$\begin{array}{cc} \varphi^{\left(n\right)}\left(k^{n}\right):= & \phi^{\left(n\right)}\left(k^{n}\right)⊕b^{m}=k^{n}A⊕b^{m},\;\mathrm{for}\;k^{n}\in X^{n}. \end{array}$$

The mapping $\varphi^{\left(n\right)}$ is called the affine mapping induced by the linear mapping $\phi^{\left(n\right)}$ and constant vector $b^{m}$$\in X^{m}$. By the definition of $\varphi^{\left(n\right)}$ shown in (3), the following *affine structure* holds:

(4) $$\begin{array}{c} \varphi^{\left(n\right)}\left(x^{n}⊕k^{n}\right)=\left(x^{n}⊕k^{n}\right)A⊕b^{m}=x^{n}A⊕\left(k^{n}A⊕b^{m}\right)=\phi^{\left(n\right)}\left(x^{n}\right)⊕\varphi^{\left(n\right)}\left(k^{n}\right),\;\mathrm{for}\;x^{n},k^{n}\in X^{n}. \end{array}$$

Next, let $\psi^{\left(n\right)}$ be the corresponding decoder for $\phi^{\left(n\right)}$ such that $\psi^{\left(n\right)}:X^{m}\to X^{n}.$ Note that $\psi^{\left(n\right)}$ does not have a linear structure in general.

*Description of Proposed Procedure:* We describe the procedure of our privacy amplified system as follows.

1. *Encoding of Ciphertext:* First, we use $\varphi^{\left(n\right)}$ to encode the ciphertext $C^{n}=X^{n}⊕K^{n}$. Let ${\tilde{C}}^{m}=\varphi^{\left(n\right)}\left(C^{n}\right)$. Then, instead of sending $C^{n}$, we send ${\tilde{C}}^{m}$ to the public communication channel. By the affine structure of the encoder $\varphi^{\left(n\right)}$ (shown in (4)) we have
  (5) $$\begin{array}{c} {\tilde{C}}^{m}=\varphi^{\left(n\right)}\left(X^{n}⊕K^{n}\right)=\phi^{\left(n\right)}\left(X^{n}\right)⊕\varphi^{\left(n\right)}\left(K^{n}\right)={\tilde{X}}^{m}⊕{\tilde{K}}^{m}, \end{array}$$
  where we set ${\tilde{X}}^{m}:=\phi^{\left(n\right)}\left(X^{n}\right),{\tilde{K}}^{m}:=\varphi^{\left(n\right)}\left(K^{n}\right).$
2. *Decoding at Sink Node*D: First, using the linear encoder $\varphi^{\left(n\right)}$, D encodes the key $K^{n}$ received through a private channel into ${\tilde{K}}^{m}=$$(\varphi^{\left(n\right)}\left(K^{n}\right)$. Receiving ${\tilde{C}}^{m}$ from the public communication channel, D computes ${\tilde{X}}^{m}$ in the following way. From (5), we have that the decoder D can obtain ${\tilde{X}}^{m}$ $=\phi^{\left(n\right)}\left(X^{n}\right)$ by subtracting ${\tilde{K}}^{m}=\varphi^{\left(n\right)}\left(K^{n}\right)$ from ${\tilde{C}}^{m}$. Finally, D outputs ${\hat{X}}^{n}$ by applying the decoder $\psi^{\left(n\right)}$ to ${\tilde{X}}^{m}$ as follows:
  (6) $$\begin{array}{cc} {\hat{X}}^{n} & =\psi^{\left(n\right)}\left({\tilde{X}}^{m}\right)=\psi^{\left(n\right)}\left(\phi^{\left(n\right)}\left(X^{n}\right)\right). \end{array}$$

Our concrete privacy-amplified system described above is illustrated in Figure 4.

### Splitting of Reliability and Security

**Figure 5:**
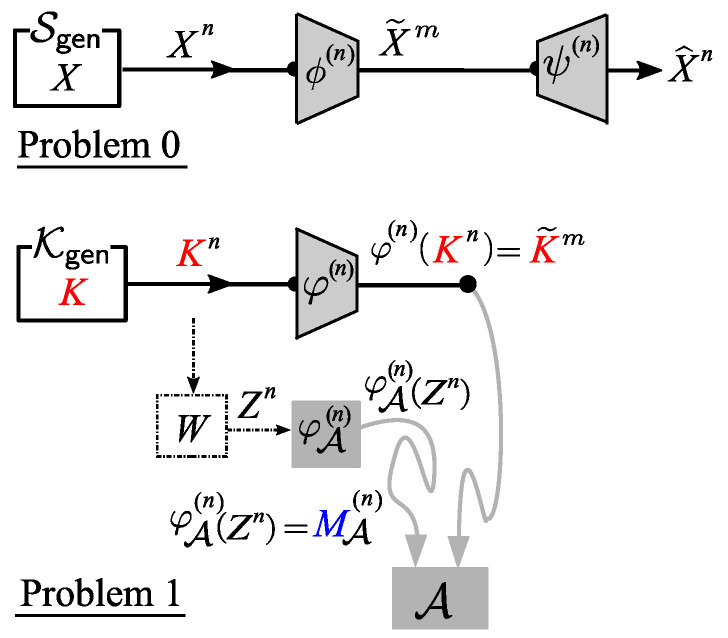
Two split problems: Problem 0 (Reliability) and Problem 1 (Security).

By the affine structure of the encoder function $\varphi^{\left(n\right)}$, the proposed privacy amplified system can be split into two coding problems. One is a source coding problem using a linear encoder $\phi^{\left(n\right)}$. We hereafter call this Problem 0. The other is a privacy amplification problem using the affine encoder $\varphi^{\left(n\right)}$. We call this Problem 1. These two problems are shown in Figure 5.

*On Reliability (Problem 0):* From the description of our system in the previous section, the decoding process in our system above is successful if ${\hat{X}}^{n}=X^{n}$ holds. Combining this and (6), it is clear that the decoding error probability $p_{e}$ is as follows:

$$\begin{array}{cc} p_{e}= & p_{e}\left(\varphi^{\left(n\right)},\psi^{\left(n\right)}|p_{X}^{n}\right)=\mathrm{Pr}\left[\psi^{\left(n\right)}\left(\phi^{\left(n\right)}\left(X^{n}\right)\right)\neq X^{n}\right]. \end{array}$$

In Problem 0, we discuss the minimum rate *R* such that $\exists \{(\phi^{\left(n\right)},$$\psi^{\left(n\right)}{)\}}_{n\geq 1}$ such that ∀ϵ>0, $\exists n_{0}=n_{0}\left(\epsilon \right)\in N_{0}$, $\forall n\geq n_{0}$, we have

$$\begin{array}{c} \frac{1}{n}\log|X^{m}|=\frac{m}{n}\log\left|X\right|\leq R+\varepsilon ,p_{e}\left(\phi^{\left(n\right)},\psi^{\left(n\right)}|p_{X}^{n}\right)\leq \epsilon . \end{array}$$

It is well known that this minimum is equal to H(X) when ${\left\{\phi^{\left(n\right)}\right\}}_{n\geq }$ is a sequence of general (nonlinear) encoders. Csiszár [6] proved the existence of a sequence of linear encoders and nonlinear decoders $\{(\phi^{\left(n\right)},$$\psi^{\left(n\right)}{)\}}_{n\geq 1}$ such that for any $p_{X}$ satisfying R>H(X), the error probability $p_{e}\left(\phi^{\left(n\right)},\psi^{\left(n\right)}|p_{X}^{n}\right)$ decays exponentially as n→∞. His result is stated in the next section.

*On Security (Problem 1):* We assume that the adversary A knows $\left(A,b^{n}\right)$ defining the affine encoder $\varphi^{\left(n\right)}$. When $\varphi^{\left(n\right)}$ has the affine structure shown in (4), the information leakage $\Delta^{\left(n\right)}$ measured by the mutual information between $X^{n}$ and $({\tilde{C}}^{m},$ $M_{A}^{\left(n\right)})$ has the following form:

(7) $$\begin{array}{c} \Delta^{\left(n\right)}=\Delta^{\left(n\right)}\left(\varphi^{\left(n\right)},\varphi_{A}^{\left(n\right)}|p_{X}^{n},p_{K}^{n},W^{n}\right)=I\left(X^{n};{\tilde{C}}^{m},M_{A}^{\left(n\right)}\right)=I\left(X^{n};\varphi^{\left(n\right)}\left(X^{n}⊕K^{n}\right),M_{A}^{\left(n\right)}\right),\\ \overset{\left(a\right)}{=}I\left(X^{n};\varphi^{\left(n\right)}\left(X^{n}\right)⊕\phi^{\left(n\right)}\left(K^{n}\right),M_{A}^{\left(n\right)}\right)=I\left(X^{n};{\tilde{X}}^{m}⊕{\tilde{K}}^{m}|M_{A}^{\left(n\right)}\right). \end{array}$$

Step (a) follows from $X_{1}^{n}⊥M_{A}^{\left(n\right)}$. Using (7), we upper bound $\Delta^{\left(n\right)}=I\left(X^{n};{\tilde{C}}^{m},M_{A}^{\left(n\right)}\right)$ to obtain the following lemma.

**Lemma** **1.** (8) $$\begin{array}{c} \Delta^{\left(n\right)}=I\left(X^{n};{\tilde{C}}^{m},M_{A}^{\left(n\right)}\right)\leq \left.\left.\left.\;\;\;D\left(p_{{\tilde{K}}^{m}|M_{A}^{\left(n\right)}}\right|\right|p_{V^{m}}\right|p_{M_{A}^{\left(n\right)}}\right), \end{array}$$

*where*$p_{V^{m}}$*represents the uniform distribution over*$X^{m}$.

**Proof.**  We have the following chain of inequalities:

$$\begin{array}{c} \Delta^{\left(n\right)}=I\left(X^{n};{\tilde{C}}^{m},M_{A}^{\left(n\right)}\right)\overset{\left(a\right)}{=}I\left(X_{1}^{n};{\tilde{X}}^{m}+{\tilde{K}}^{m}|M_{A}^{\left(n\right)}\right)\leq \log\left|X^{m}\right|-H\left({\tilde{X}}^{m}+{\tilde{K}}^{m}|X^{n},M_{A}^{\left(n\right)}\right)\\ \overset{\left(b\right)}{=}\log|X^{m}|-H\left({\tilde{K}}^{m}|X^{n},M_{A}^{\left(n\right)}\right)\overset{\left(c\right)}{=}\log\left|X^{m}\right|-H\left({\tilde{K}}^{m}|M_{A}^{\left(n\right)}\right)=\left.\left.\left.\;\;D\left(p_{{\tilde{K}}^{m}|M_{A}^{\left(n\right)}}\right|\right|p_{V^{m}}\right|p_{M_{A}^{\left(n\right)}}\right). \end{array}$$

Step (a) follows from (7). Step (b) follows from ${\tilde{X}}^{m}=\phi^{\left(n\right)}\left(X^{n}\right)$. Step (c) follows from $\left({\tilde{K}}^{m},M_{A}^{\left(n\right)}\right)⊥X_{1}^{n}$. □

We set

$$\xi_{D}^{\left(n\right)}=\xi_{D}^{\left(n\right)}\left(\varphi^{\left(n\right)},R_{A}|p_{K}^{n},W^{n}\right):=\max_{\varphi_{A}^{\left(n\right)}\in F^{\left(n\right)}\left(R_{A}\right)}\left.\left.\left.\;\;D\left(p_{{\tilde{K}}^{m}|M_{A}^{\left(n\right)}}\right|\right|p_{V^{m}}\right|p_{M_{A}^{\left(n\right)}}\right).$$

Then we have the following lemma.

**Lemma** **2.** *For any affine encoder*$\varphi^{\left(n\right)}:X^{n}\to X^{m}$, *we have*

$$\Delta^{\left(n\right)}\left(\varphi^{\left(n\right)},\varphi_{A}^{\left(n\right)}|p_{X}^{n},p_{K}^{n},W^{n}\right)\leq \xi_{D}^{\left(n\right)}\left(\varphi^{\left(n\right)},R_{A}|p_{K}^{n},W^{n}\right).$$

The quantity $\xi_{D}^{\left(n\right)}\left(\varphi^{\left(n\right)},R_{A}|p_{K}^{n},W^{n}\right)$ will play an important role in deriving an explicit upper bound of $\Delta^{\left(n\right)}\left(\varphi^{\left(n\right)},\varphi_{A}^{\left(n\right)}|p_{X}^{n},p_{K}^{n},W^{n}\right).$ In Problem 1, we consider the privacy amplification problem using the quantity $\xi_{D}^{\left(n\right)}\left(\varphi^{\left(n\right)},R_{A}|p_{K}^{n},W^{n}\right)$ as a security criterion. In this problem, we study an explicit characterization of the region denoted by $R_{P1}\left(p_{K},W\right)$, which consists of all pairs $\left(R,R_{A}\right)$ such that $\exists {\left\{\varphi^{\left(n\right)}\right\}}_{n\geq 1}$ such that $\forall \varepsilon >0,\exists n_{0}=n_{0}\left(\varepsilon \right)\in N_{0},\forall n\geq n_{0},$

$$\frac{1}{n}\log||\varphi^{\left(n\right)}\left|\right|=\frac{m}{n}\log\left|X\right|\geq R-\varepsilon and\xi_{D}^{\left(n\right)}\left(\varphi^{\left(n\right)},R_{A}|p_{K}^{n},W^{n}\right)\leq \varepsilon .$$

In the next section, we discuss two previous works related to Problem 1.

## 4. Previous Related Works

In this section, we introduce approaches from previous existing work related to Problem 0 (reliability) and Problem 1 (security). Our goal is that by showing these previous approaches, it will be easier to understand our approach to analyzing reliability and security. In particular, for Problem 1 (security), we explain approaches used in similar problems in previous works and highlight their differences from Problem 1.

We first state a previous result related to Problem 0. Let $\varphi^{\left(n\right)}$ be an affine encoder and $\phi^{\left(n\right)}$ be a linear encoder induced by $\varphi^{\left(n\right)}$. We define a function related to an exponential upper bound of $p_{e}\left(\phi^{\left(n\right)},\psi^{\left(n\right)}|p_{X}^{n}\right)$. Let $\bar{X}$ be an arbitrary random variable over X that has a probability distribution $p_{\bar{X}}$. Let P(X) denote the set of all probability distributions on X. For R≥0 and $p_{X}\in$P(X), we define the following function:

$$\begin{array}{cc} E(R|p_{X}) & :=\min_{p_{\bar{X}}\in P\left(X\right)}\{{\left[R-H\left(\bar{X}\right)\right]}^{+}+D(p_{\bar{X}}\left|\right|p_{X})\}. \end{array}$$

By simple computation, we can prove that $E(R|p_{X})$ takes positive values if and only if R>H(X). We have the following result.

**Theorem** **1.**  (Csiszár [6]). *There exists a sequence* $\{{\left(\phi^{\left(n\right)},\psi^{\left(n\right)}\right\}}_{n\geq 1}$ *such that for any* $p_{X}$, *we have*

(9) $$\begin{array}{c} \frac{1}{n}\log|X^{m}|=\frac{m}{n}\log\left|X\right|\leq R,p_{e}\left(\phi^{\left(n\right)},\psi^{\left(n\right)}|p_{X}^{n}\right)\leq e^{-n[E\left(R|p_{X}\right)-\delta_{n}]}, \end{array}$$

*where* $\delta_{n}$ *is defined by*

$$\begin{array}{c} \delta_{n}:=\frac{1}{n}\log\left[e{\left(n+1\right)}^{3|X|}\right]. \end{array}$$

*Note that* $\delta_{n}\to 0$ *as* n→∞.

It follows from Theorem 1 that if R>H(X), then the error probability of decoding $p_{e}\left(\phi^{\left(n\right)},\psi^{\left(n\right)}|p_{X}^{n}\right)$ decays exponentially, and its exponent is lower bounded by the quantity $E(R|p_{X})$. Furthermore, the code ${\left\{\left(\phi^{\left(n\right)},\psi^{\left(n\right)}\right)\right\}}_{n\geq 1}$ is a universal code that depends only on the rate *R* and not on the value of $p_{X}\in P\left(X\right)$.

**Figure 6:**
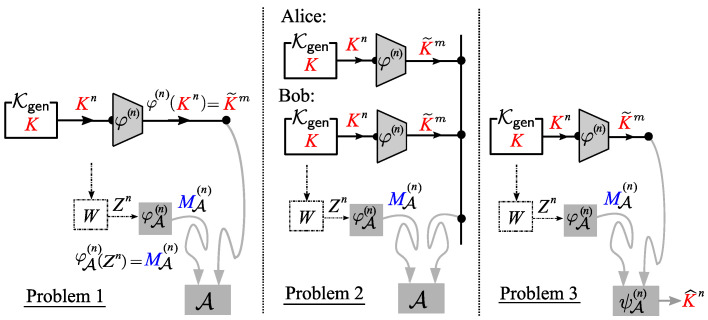
Three related coding problems.

We next state two coding problems related to Problem 1. One is a problem on the privacy amplification for the bounded storage eavesdropper posed and investigated by Watanabe and Oohama [10]. The other is the one helper source coding problem posed and investigated by Ashlswede and Körner [7] and Wyner [16]. We hereafter call the former and latter problems, respectively, Problem 2 and Problem 3. Problems 1–3 are shown in Figure 6. As we can see from this figure, these three problems are based on the same communication scheme. The classes of encoder functions and the security criteria on A are different between these three problems. In Problem 1, the sequence of encoding functions ${\left\{\varphi^{\left(n\right)}\right\}}_{n\geq 1}$ is restricted to the class of affine encoders to satisfy the homomorphic property. On the other hand, in Problems 2 and 3, we have no such restriction on the class of encoder functions. In descriptions of Problems 2 and 3, we state the difference in security criteria between Problems 1, 2, and 3. A comparison of three problems in terms of ${\left\{\varphi^{\left(n\right)}\right\}}_{n\geq 1}$ and security criteria is summarized in Table 1.

In Problem 2, Alice and Bob share a random variable $K^{n}$ of block length *n*, and an eavesdropper adversary A has a random variable $Z^{n}$ that is correlated to $K^{n}$. In such a situation, Alice and Bob try to distill a secret key as long as possible. In [10], they considered a situation such that the adversary’s random variable $Z^{n}$ is stored in a storage that is obtained as a function value of $Z^{n}$, and the rate of the storage size is bounded. This situation makes sense when the alphabet size of the adversary’s observation $Z^{n}$ is too huge to be stored directly in a storage. In such a situation, Watanabe and Oohama [10] obtained an explicit characterization of the region $R_{\mathrm{WO}}\left(p_{K},W\right)$ indicating the trade-off between the key rate R=(m/n)log|X| and the rate $R_{A}=\left(1/n\right)\log|M_{A}^{\left(n\right)}$ of the storage size. In Problem 2, the variational distance $d(p_{V^{m}}\times p_{M_{A}^{\left(n\right)}},p_{{\tilde{K}}^{m}M_{A}^{\left(n\right)}})$ between $p_{V^{m}}\times p_{M_{A}^{\left(n\right)}}$ and $p_{{\tilde{K}}^{m}M_{A}^{\left(n\right)}})$ is used as a security criterion instead of $D(p_{{\tilde{K}}^{m}|M_{A}^{\left(n\right)}}||p_{V^{m}}|p_{M_{A}^{\left(n\right)}})$ in Problem 1. Define

$$\xi_{d}^{\left(n\right)}=\xi_{d}^{\left(n\right)}\left(\varphi^{\left(n\right)},R_{A}|p_{K}^{n},W^{n}\right):=\max_{\varphi_{A}^{\left(n\right)}\in F^{\left(n\right)}\left(R_{A}\right)}d\left(p_{V^{m}}\times p_{M_{A}^{\left(n\right)}},p_{{\tilde{K}}^{m}M_{A}^{\left(n\right)}}\right).$$

Then the formal definition of the region $R_{\mathrm{WO}}\left(p_{K},W\right)$ is given by the following:

$$\begin{array}{cc} R_{\mathrm{WO}}\left(p_{K},W\right):= & \left\{(R_{A},R\right):\exists {\left\{\varphi^{\left(n\right)}\right\}}_{n\geq 1}\;\mathrm{such}\;\mathrm{that}\;\forall \varepsilon >0,\exists n_{0}=n_{0}\left(\varepsilon \right)\in N_{0},\forall n\geq n_{0},\\ & \left(m/n\right)\log|X|\geq R-\varepsilon \;\mathrm{and}\;\xi_{d}^{\left(n\right)}\left(\varphi^{\left(n\right)},R_{A}|p_{K}^{n},W^{n}\right)\leq \varepsilon \}. \end{array}$$

In Problem 3, the adversary outputs an estimation ${\hat{K}}^{n}$ of $K^{n}$ from ${\tilde{K}}^{m}=\varphi^{\left(n\right)}\left(K^{n}\right)$ and $M_{A}^{\left(n\right)}=\varphi_{A}^{\left(n\right)}\left(Z^{n}\right)$. Let $\psi_{A}^{\left(n\right)}:M^{\left(n\right)}\times X^{m}$ be a decoder function of the adversary. Then ${\hat{K}}^{n}$ is given by ${\hat{K}}^{n}=\psi_{A}^{\left(n\right)}(\varphi_{A}^{\left(n\right)}\left(Z^{n}\right),{\tilde{K}}^{m}=\varphi^{\left(n\right)}\left(K^{n}\right).$ Let

$$p_{e,A}^{\left(n\right)}=p_{e,A}^{\left(n\right)}\left(\varphi^{\left(n\right)},\varphi_{A}^{\left(n\right)}\psi_{A}^{\left(n\right)}|p_{K}^{n},W^{n}\right):=\mathrm{Pr}\left\{K^{n}\neq \psi_{A}^{\left(n\right)}\left(\varphi_{A}^{\left(n\right)}\left(Z^{n}\right),\varphi^{\left(n\right)}\left(K^{n}\right)\right)\right\}$$

be the error probability of decoding for Problem 3. The quantity $M_{A}^{\left(n\right)}$ serves as a helper for the decoding of $K^{n}$ from ${\tilde{K}}^{m}$. In Problem 3, Ahlswede and Körner [7] and Wyner [16] investigated an explicit characterization of the rate region $R_{\mathrm{AKW}}\left(p_{K},W\right)$ indicating the trade-off between $R_{A}$ and *R* under the condition that $p_{e,A}^{\left(n\right)}=\mathrm{Pr}\left\{K^{n}\neq {\hat{K}}_{n}\right\}$ vanishes asymptotically. The region $R_{\mathrm{AKW}}\left(p_{K},W\right)$ is formally defined by

$$\begin{array}{cc} R_{\mathrm{AKW}}\left(p_{K},W\right):= & \left\{(R_{A},R\right):\exists \{{\left(\varphi^{\left(n\right)},\varphi_{A}^{\left(n\right)},\psi_{A}^{\left(n\right)}\right\}}_{n\geq 1}\;\mathrm{such}\;\mathrm{that}\\ & \forall \varepsilon >0,\exists n_{0}=n_{0}\left(\varepsilon \right)\in N_{0},\forall n\geq n_{0},\\ & \left(m/n\right)\log|X|\leq R+\varepsilon ,\varphi_{A}^{\left(n\right)}\in F_{A}\left(R+\varepsilon \right),\\ & \mathrm{and}\;p_{e,A}^{\left(n\right)}\left(\varphi^{\left(n\right)},\varphi_{A}^{\left(n\right)},\psi_{A}^{\left(n\right)}|p_{K}^{n},W^{n}\right)\leq \varepsilon \}. \end{array}$$

The region $R_{\mathrm{AKW}}\left(p_{K},W\right)$ was determined by Ashlswede and Körner [7] and Wyner [16]. To state their result, we define several quantities. Let U be an auxiliary random variable taking values in a finite set U. We assume that the joint distribution of (U,Z,K) is

$$p_{UZK}\left(u,z,k\right)=p_{U}\left(u\right)p_{Z|U}\left(z|u\right)p_{K|Z}\left(k|z\right).$$

The above condition is equivalent to U↔Z↔K. Define the set of probability distribution $p=p_{UZK}$ by

$$\begin{array}{c} P\left(p_{K},W\right):=\{p_{UZK}:|U|\leq |Z|+1,U↔Z↔K\}. \end{array}$$

Set

$$\begin{array}{cc} R(p):= & \begin{array}{c} \{\left(R_{A},R\right):R_{A},R\geq 0,R_{A}\geq I\left(Z;U\right),R\geq H\left(K|U\right)\}, \end{array}\\ R(p_{K},W):= & \underset{p\in P(p_{K},W)}{⋃}R\left(p\right). \end{array}$$

We can show that the region $R(p_{K},W)$ satisfies the following property.

**Propert**y **1.**

*(a)*  *The region*$R(p_{K},W)$*is a closed convex subset of*$R_{+}^{2}:=\left\{R_{A}\geq 0,R\geq 0\right\}$.
*(b)*  *For any*$\left(p_{K},W\right)$, *we have*
  (10) $$\min_{\left(R_{A},R\right)\in R\left(p_{K},W\right)}\left(R_{A}+R\right)=H\left(K\right).$$
  *The minimum is attained by*$\left(R_{A},R\right)=(0,H(K$)). *This result implies that*
  $$\begin{array}{cc} R(p_{K},W)\subseteq & \left\{\left(R_{A},R\right):R_{A}+R\geq H\left(K\right)\right\}\cap R_{+}^{2}. \end{array}$$
  *Furthermore, the point*(0,H(K))*always belongs to*$R(p_{K},W)$.

**Figure 7:**
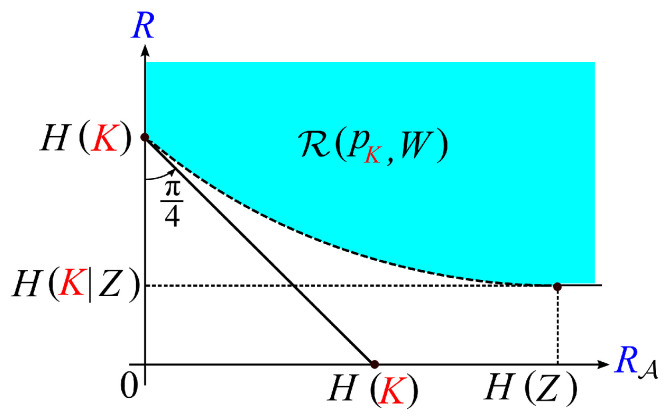
Shape of the region $R(p_{K},W)$.

Property 1 part (a) is a well-known property. Proof of Property 1 part (b) is easy. Proofs of Property 1 parts (a) and (b) are omitted. Typical shape of the region $R(p_{K},W)$ is shown in Figure 7.

The rate region $R_{\mathrm{AKW}}\left(p_{K},W\right)$ was determined by Ahlswede and Körner [7] and Wyner [16]. Their result is the following.

**Theorem** **2.** (Ahlswede, Körner [7] andWyner [16])

$$\begin{array}{c} R_{\mathrm{AKW}}\left(p_{K},W\right)=R\left(p_{K},W\right). \end{array}$$

Watanabe and Oohama [10] investigated an explicit form of $R_{\mathrm{WO}}\left(p_{K},W\right)$ to show that it is equal to $R^{c}\left(p_{K},W\right)$, that is, we have the following result.

**Theorem** **3.** (Watanabe and Oohama [10])

$$\begin{array}{c} R_{\mathrm{WO}}\left(p_{K},W\right)=R_{\mathrm{AKW}}^{c}\left(p_{K},W\right)=R^{c}\left(p_{K},W\right). \end{array}$$

In the remaining part of this section, we investigate a relationship between Problems 2 and 3 to give an outline of the proof of this theorem. Let

$$p_{c,A}^{\left(n\right)}=p_{c,A}^{\left(n\right)}\left(\varphi^{\left(n\right)},\varphi_{A}^{\left(n\right)},\psi_{A}^{\left(n\right)}|p_{K}^{n},W^{n}\right):=\mathrm{Pr}\left\{K^{n}=\psi_{A}^{\left(n\right)}\left(\varphi_{A}^{\left(n\right)}\left(Z^{n}\right),\varphi^{\left(n\right)}\left(K^{n}\right)\right)\right\}$$

be the correct probability of decoding for Problem 3. The following lemma provides an important inequality to examine a relationship between these two problems.

**Lemma** **3.** *For any*$\left(\varphi^{\left(n\right)},\varphi_{A}^{\left(n\right)},\psi_{A}^{\left(n\right)}\right)$, *we have the following:*

$$p_{c,A}^{\left(n\right)}\left(\varphi^{\left(n\right)},\varphi_{A}^{\left(n\right)},\psi_{A}^{\left(n\right)}|p_{K}^{n},W^{n}\right)\leq \frac{1}{{\left|X\right|}^{m}}+d\left(p_{V^{m}}\times p_{M_{A}^{\left(n\right)}},p_{{\tilde{K}}^{m}M_{A}^{\left(n\right)}}\right).$$

Proof of this lemma is given in Appendix A. Using Lemma 3, we can easily prove the inclusion $R_{\mathrm{WO}}\left(p_{K},W\right)\subseteq R_{\mathrm{AKW}}\left(p_{K},W\right)$, which corresponds to the converse part of Theorem 3.

**Proof of**$R_{\mathrm{WO}}\left(p_{K},W\right)\subseteq R_{\mathrm{AKW}}^{c}\left(p_{K},W\right)$: We assume that $\left(R_{A},R\right)\in R_{\mathrm{AKW}}\left(p_{K},W\right)$. Then there exists $\{{\left(\varphi^{\left(n\right)},\varphi_{A}^{\left(n\right)},\psi_{A}^{\left(n\right)}\right\}}_{n\geq 1}$ such that ∀ε>0, $\exists n_{0}=n_{0}\left(\varepsilon \right)\in N_{0},$ $\forall n\geq n_{0}$,

(11) $$\begin{array}{c} \frac{m}{n}\log\left|X\right|\leq R+\varepsilon ,\varphi_{A}^{\left(n\right)}\in F_{A}^{\left(n\right)}\left(R+\varepsilon \right), \end{array}$$

(12) $$\begin{array}{c} \mathrm{and}\;p_{e,A}^{\left(n\right)}\left(\varphi^{\left(n\right)},\varphi_{A}^{\left(n\right)},\psi_{A}^{\left(n\right)}|p_{K}^{n},W^{n}\right)\leq \varepsilon . \end{array}$$

From the above sequence ${\left\{\left(\varphi^{\left(n\right)},\varphi_{A}^{\left(n\right)},\psi_{A}^{\left(n\right)}\right)\right\}}_{n\geq 1}$, we can construct the sequence $\{{\left({\hat{\varphi }}^{\left(n\right)},\varphi_{A}^{\left(n\right)},\psi_{A}^{\left(n\right)}\right\}}_{n\geq 1}$ such that

(13) $$\begin{array}{c} R+\varepsilon \geq \frac{1}{n}\log||{\hat{\varphi }}^{\left(n\right)}\left|\right|=\frac{\hat{m}}{n}\log\left|X\right|\geq \max\left\{R-\varepsilon ,\frac{m}{n}\log\left|X\right|\right\},\;\varphi_{A}^{\left(n\right)}\in F_{A}^{\left(n\right)}\left(R+\varepsilon \right), \end{array}$$

(14) $$\begin{array}{c} p_{e,A}^{\left(n\right)}\left({\hat{\varphi }}^{\left(n\right)},\varphi_{A}^{\left(n\right)},\psi_{A}^{\left(n\right)}|p_{K}^{n},W^{n}\right)\leq p_{e,A}^{\left(n\right)}\left(\varphi^{\left(n\right)},\varphi_{A}^{\left(n\right)},\psi_{A}^{\left(n\right)}|p_{K}^{n},W^{n}\right)\leq \varepsilon . \end{array}$$

Set ${\tilde{K}}^{\hat{m}}:={\hat{\varphi }}^{\left(n\right)}\left(K^{n}\right)$. Then from (14) and Lemma 3, we have

$$\begin{array}{c} d\left(p_{V^{\hat{m}}}\times p_{M_{A}^{\left(n\right)}},p_{{\tilde{K}}^{\hat{m}}M_{A}^{\left(n\right)}}\right)\geq 1-\varepsilon -\frac{1}{{\left|X\right|}^{\hat{m}}}, \end{array}$$

from which we have

(15) $$\begin{array}{c} d\left(p_{V^{\hat{m}}}\times p_{M_{A}^{\left(n\right)}},p_{{\tilde{K}}^{\hat{m}}M_{A}^{\left(n\right)}}\right)\geq 1-2\varepsilon , \end{array}$$

for sufficiently large *n*. From (13), (15), and the definition of $R_{\mathrm{WO}}\left(p_{K},W\right)$, we can see that $\left(R_{A}+\varepsilon ,R\right)\notin R_{\mathrm{WO}}\left(p_{K},W\right)$, or equivalent to

(16) $$\begin{array}{c} \left(R_{A}+\varepsilon ,R\right)\in R_{\mathrm{WO}}^{c}\left(p_{K},W\right)\Leftrightarrow \left(R_{A},R\right)\in R_{\mathrm{WO}}^{c}\left(p_{K},W\right)-\varepsilon \left(1,0\right), \end{array}$$

where we set R−(a,b):={(u,v):(u+a,v+b)∈R}. Since $\left(R_{A},R\right)\in R_{\mathrm{AKW}}\left(p_{K},W\right)$ is arbitrary, we have that

(17) $$\begin{array}{c} R_{\mathrm{AKW}}\left(p_{K},W\right)\subseteq R_{\mathrm{WO}}^{c}\left(p_{K},W\right)-\varepsilon \left(1,0\right)\Leftrightarrow R_{\mathrm{AKW}}\left(p_{K},W\right)+\varepsilon \left(1,0\right)\subseteq R_{\mathrm{WO}}^{c}\left(p_{K},W\right)\\ \Leftrightarrow R_{\mathrm{WO}}\left(p_{K},W\right)\subseteq R_{\mathrm{AKW}}^{c}\left(p_{K},W\right)+\varepsilon \left(1,0\right)\Leftrightarrow R_{\mathrm{WO}}\left(p_{K},W\right)\subseteq R^{c}\left(p_{K},W\right)+\varepsilon \left(1,0\right). \end{array}$$

By letting ε→0 in (17) and considering that $R^{c}\left(p_{K},W\right)$ is an open set, we have that $R_{\mathrm{WO}}\left(p_{K},W\right)\subseteq R^{c}\left(p_{K},W\right)$. □

To prove $R_{\mathrm{WO}}\left(p_{K},W\right)⊇R_{\mathrm{AKW}}^{c}$, we examine an upper bound of $\xi_{d}^{\left(n\right)}\left(\varphi^{\left(n\right)},R_{A}|p_{K}^{n},W^{n}\right)$. For η>0, we define

$$\begin{array}{c} {℘}_{\eta }^{\left(n\right)}={℘}_{\eta }^{\left(n\right)}\left(R|p_{K}^{n},W^{n}\right):=p_{M_{A}^{\left(n\right)}Z^{n}K^{n}}\left\{R\geq \frac{1}{n}\log\frac{1}{p_{K^{n}|M_{A}^{\left(n\right)}}\left(K^{n}|M_{A}^{\left(n\right)}\right)}-\eta \right\},\\ \Phi_{d,\eta }^{\left(n\right)}\left(R_{A},R|p_{K}^{n}W^{n}\right):=\max_{\varphi_{A}^{\left(n\right)}\in F^{\left(n\right)}\left(R_{A}\right)}\left\{{℘}_{\eta }^{\left(n\right)}\left(R|p_{K}^{n},W^{n}\right)+\sqrt{e^{-n\eta }}\right\}. \end{array}$$

According to Watanabe and Oohama [10], we have the following two propositions.

**Proposition** **1.** (Watanabe and Oohama [10]). *Fix any positive η>0. $\exists \varphi^{\left(n\right)}:X^{n}\to X^{m}$ satisfying (m/n)log|X|≥R−2η, we have*

$$\xi_{d}^{\left(n\right)}\left(\varphi^{\left(n\right)},R_{A}|p_{K}^{n},W^{n}\right)\leq \Phi_{d,\eta }^{\left(n\right)}\left(R_{A},R|p_{K}^{n}W^{n}\right).$$

**Proposition** **2.** (Watanabe and Oohama [10]). *If* $\left(R_{A},R\right)\notin R\left(p_{K},W\right)$, *then for any* η>0 *and any* $\varphi_{A}^{\left(n\right)}\in F_{A}^{\left(n\right)}\left(R_{A}\right)$, *we have*

$$\lim_{n\to \infty }{℘}_{\eta }^{\left(n\right)}\left(R|p_{K}^{n},W\right)=0,$$

*which implies that*

$$\lim_{n\to \infty }\Phi_{d,\eta }^{\left(n\right)}\left(R_{A},R|p_{K}^{n}W^{n}\right)=0.$$

The inclusion $R_{\mathrm{WO}}\left(p_{K},W\right)⊇R_{\mathrm{AKW}}^{c}$ immediately follows from Propositions 1 and 2.

## 5. Reliability and Security Analysis

In this section, we state our main results. We use the affine encoder $\varphi^{\left(n\right)}$ defined in the previous section. We upper bound $p_{e}=p_{e}\left(\varphi^{\left(n\right)},\psi^{\left(n\right)}|p_{X}^{n}\right)$ and $\Delta^{\left(n\right)}=\Delta^{\left(n\right)}\left(\varphi^{\left(n\right)},\varphi_{A}^{\left(n\right)}|p_{X}^{n},p_{K}^{n},W^{n}\right)$ to obtain inner bounds of $R_{\mathrm{Sys}}(p_{X},$$p_{K},W)$ and $D_{\mathrm{Sys}}(p_{X},$$p_{K},W)$.

Let

$$\begin{array}{c} \Phi_{D,\eta }^{\left(n\right)}\left(R_{A},R|p_{K}^{n}W^{n}\right):=\max_{\varphi_{A}^{\left(n\right)}\in F^{\left(n\right)}\left(R_{A}\right)}\left\{nR{℘}_{\eta }^{\left(n\right)}\left(R|p_{K}^{n},W^{n}\right)+e^{-n\eta }\right\},\\ \Phi_{D}^{\left(n\right)}\left(R_{A},R|p_{K}^{n}W^{n}\right):=\underset{\eta >0}{\inf}\Phi_{D,\eta }\left(R_{A},R|p_{K}^{n}W^{n}\right). \end{array}$$

Then we have the following proposition.

**Proposition** **3.** *For any*$R_{A},R>0$*and any*$\left(p_{K},W\right)$, *there exists a sequence of mappings*${\left\{\left(\varphi^{\left(n\right)},\psi^{\left(n\right)}\right)\right\}}_{n=1}^{\infty }$*such that for any*$p_{X}\in P\left(X\right)$, *we have*

(18) $$\begin{array}{c} R-\frac{1}{n}\leq \frac{1}{n}\log|X^{m}|=\frac{m}{n}\log\left|X\right|\leq R,\\ p_{e}\left(\phi^{\left(n\right)},\psi^{\left(n\right)}|p_{X}^{n}\right)\leq e{\left(n+1\right)}^{2|X|}\left\{{\left(n+1\right)}^{\left|X\right|}+1\right\}e^{-nE(R|p_{X})}, \end{array}$$

*and for any eavesdropper*A*with*$\varphi_{A}$*satisfying*$\varphi_{A}^{\left(n\right)}\in F_{A}^{\left(n\right)}\left(R_{A}\right)$, *we have*

(19) $$\begin{array}{c} \Delta^{\left(n\right)}\left(\varphi^{\left(n\right)},\varphi_{A}^{\left(n\right)}|p_{X}^{n},p_{K}^{n},W^{n}\right)\leq \left\{{\left(n+1\right)}^{\left|X\right|}+1\right\}\Phi_{D}^{\left(n\right)}\left(R_{A},R|p_{K}^{n}W^{n}\right). \end{array}$$

This proposition can be proved by several tools developed by previous works. The detail of the proof is given in the next section. As we stated in Proposition 2, Watanabe and Oohama [10] proved that if $\left(R_{A},R\right)\notin R\left(p_{K},W\right)$, then the quantity for any η>0 and any $\varphi_{A}^{\left(n\right)}\in F_{A}^{\left(n\right)}\left(R_{A}\right)$, the quantity ${℘}_{\eta }^{\left(n\right)}\left(R|p_{K}^{n},W\right)$. Their method can not be applied to the analysis of $\Phi_{D}^{\left(n\right)}\left(R_{A},R|p_{K}^{n}W^{n}\right)$ since the quantity nR is multiplied with the quantity ${℘}_{\eta }^{\left(n\right)}\left(R|p_{K}^{n},W\right)$ in the definition of $\Phi_{D}^{\left(n\right)}\left(R_{A},R|p_{K}^{n}W^{n}\right)$. In this paper, we derive an upper bound of $\Phi_{D}^{\left(n\right)}\left(R_{A},R|p_{K}^{n}W^{n}\right)$ that decays exponentially as n→∞ if $\left(R_{A},R\right)\notin R\left(p_{K},W\right)$. To derive the upper bound, we use a new method that is developed by Oohama to prove strong converse theorems in multi-terminal source or channel networks [9,17,18,19,20].

We define several functions and sets to describe the upper bound of $\Phi_{D}^{\left(n\right)}\left(R_{A},R|p_{K}^{n}W^{n}\right)$. Set

$$\begin{array}{cc} Q(p_{K|Z}):= & \{q=q_{UZK}:|U|\leq |Z|,U↔Z↔K,p_{K|Z}=q_{K|Z}\}. \end{array}$$

For $\left(\mu ,\alpha \right)\in {\left[0,1\right]}^{2}$ and for $q=q_{UZK}\in Q\left(p_{K|Z}\right)$, define

ωq|pZ(μ,α)(z,k|u):=α¯logqZ(z)pZ(z)+αμlogqZ|U(z|u)pZ(z)+μ¯log1qK|U(k|u),Ω(μ,α)(q|pZ):=−logEqexp−ωq|pZ(μ,α)(Z,K|U),Ω(μ,α)(pK,W):=minq∈Q(pK|Z)Ω(μ,α)(q|pZ),F(μ,α)(μRA+μ¯R|pK,W):=Ω(μ,α)(pK,W)−α(μRA+μ¯R)2+αμ¯,F(RA,R|pK,W):=sup(μ,α)∈[0,1]2F(μ,α)(μRA+μ¯R|pK,W).

We next define a function serving as a lower bound of $F(R_{A},R_{}|p_{K},W)$. For each $p_{UZK}\in P_{\mathrm{sh}}\left(p_{K},W\right)$, define

ω˜p(μ)(z,k|u):=μlogpZ|U(z|u)pZ(z)+μ¯log1pK|U(K|U),Ω˜(μ,λ)(p):=−logEpexp−λω˜p(μ)(Z,K|U),Ω˜(μ,λ)(pK,W):=minp∈Psh(pK,W)Ω˜(μ,λ)(p).

Furthermore, set

F˜(μ,λ)(μRA+μ¯R|pK,W):=Ω˜(μ,λ)(pK,W)−λ(μRA+R)2+λ(5−μ),F˜(RA,R|pK,W):=supλ≥0,μ∈[0,1]F˜(μ,λ)(μRA+μ¯R|pK,W).

We can show that the above functions satisfy the following property.

**Property** **2.**

*(a)*  *The cardinality bound*|U|≤|Z|*in*$Q(p_{K|Z})$*is sufficient to describe the quantity*$\Omega^{\left(\mu ,\beta ,\alpha \right)}\left(p_{K},W\right)$. *Furthermore, the cardinality bound*|U|≤|Z|*in*$P_{\mathrm{sh}}\left(p_{K},W\right)$*is sufficient to describe the quantity*${\tilde{\Omega }}^{\left(\mu ,\lambda \right)}\left(p_{K},W\right)$.
*(b)*  *For any*$R_{A},R_{}\geq 0$, *we have*
  $$\begin{array}{c} F\left(R_{A},R_{}|p_{K},W\right)\geq \tilde{F}\left(R_{A},R_{}|p_{K},W\right). \end{array}$$
*(c)*  *For any*$p=p_{UZK}\in P_{\mathrm{sh}}\left(p_{Z},W\right)$*and any*(μ,λ)∈[0,${1]}^{2}$, *we have*
  (20) $$0\leq {\tilde{\Omega }}^{\left(\mu ,\lambda \right)}\left(p\right)\leq \mu \log|Z|+\bar{\mu }\log\left|K\right|.$$
*(d)*  *Fix any*$p=p_{UZK}\in P_{\mathrm{sh}}\left(p_{K},W\right)$*and*μ∈[0,1]. *For*λ∈[0,1], *we define a probability distribution*$p^{\left(\lambda \right)}=p_{UZK}^{\left(\lambda \right)}$*by*
  $$\begin{array}{c} p^{\left(\lambda \right)}\left(u,z,k\right):=\frac{p\left(u,z,k\right)\exp\left\{-\lambda {\tilde{\omega }}_{p}^{\left(\mu \right)}\left(z,k|u\right)\right\}}{E_{p}\left[\exp\left\{-\lambda {\tilde{\omega }}_{p}^{\left(\mu \right)}\left(Z,K|U\right)\right\}\right]}. \end{array}$$
  *Then for*λ∈[0,1/2], ${\tilde{\Omega }}^{\left(\mu ,\lambda \right)}\left(p\right)$*is twice differentiable. Furthermore, for*λ∈[0,1/2], *we have*
  $$\begin{array}{c} \frac{d}{d\lambda }{\tilde{\Omega }}^{\left(\mu ,\lambda \right)}\left(p\right)=E_{p^{\left(\lambda \right)}}\left[{\tilde{\omega }}_{p}^{\left(\mu \right)}\left(Z,K|U\right)\right],\;\frac{d^{2}}{d\lambda^{2}}{\tilde{\Omega }}^{\left(\mu ,\lambda \right)}\left(p\right)=-{\mathrm{Var}}_{p^{\left(\lambda \right)}}\left[{\tilde{\omega }}_{p}^{\left(\mu \right)}\left(Z,K|U\right)\right]. \end{array}$$
  *The second equality implies that*${\tilde{\Omega }}^{\left(\mu ,\lambda \right)}\left(p\right|p_{K}$,W)*is a concave function of*λ≥0.
*(e)*  *For*(μ,λ)∈[0,1]×[0,1/2], *define*
  ρ(μ,λ)(pK,W):=max(ν,p)∈[0,λ]×Psh(pK,W):Ω˜(μ,λ)(p)=Ω˜(μ,λ)(pK,W)Varp(ν)ω˜p(μ)(Z,K|U),
  *and set*
  $$\begin{array}{c} \rho =\rho \left(p_{K},W\right):=\max_{\left(\mu ,\lambda )\in [0,1]\times [0,1/2\right]}\rho^{\left(\mu ,\lambda \right)}\left(p_{K},W\right). \end{array}$$
  *Then we have*$\rho (p_{K},W)<\infty$. *Furthermore, for any*(μ,λ)∈[0,1]×[0,1/2], *we have*
  $${\tilde{\Omega }}^{\left(\mu ,\lambda \right)}\left(p_{K},W\right)\geq \lambda R^{\left(\mu \right)}\left(p_{K},W\right)-\frac{\lambda^{2}}{2}\rho \left(p_{K},W\right).$$
*(f)*  *For every*$\tau \in (0,\left(1/2\right)\rho \left(p_{K},W\right))$, *the condition*$(R_{A},$$R_{}+\tau )\notin R\left(p_{K},W\right)$*implies*
  $$\begin{array}{c} \tilde{F}\left(R_{A},R_{}|p_{K},W\right)>\frac{\rho (p_{K},W)}{4}\cdot g^{2}\left(\frac{\tau }{\rho (p_{K},W)}\right)>0, \end{array}$$
  *where g is the inverse function of*$\vartheta \left(a\right):=a+\left(5/4\right)a^{2},a\;\geq 0$.

Proof of this property is found in Oohama [9] (extended version). On the upper bound of $\Phi_{D}^{\left(n\right)}\left(R_{A},R|p_{K}^{n}W^{n}\right)$, we have the following:

**Proposition** **4.** *For any*n≥1/R, *we have*

(21) $$\Phi_{D}^{\left(n\right)}\left(R_{A},R|p_{K}^{n}W^{n}\right)\leq 5nRe^{-nF(R_{A},R|p_{K},W)}.$$

Proof of this proposition is given in the next section. Proposition 4 has a close connection with the one helper source coding problem, which is explained as Problem 3 in the previous section. In fact, for the proof we use the result Oohama [9] obtained for an explicit lower bound of the optimal exponent on the exponential decay of $p_{c,A}^{\left(n\right)}\left(\varphi^{\left(n\right)},\varphi_{A}^{\left(n\right)},\psi_{A}^{\left(n\right)}|p_{K}^{n},W^{n}\right)$ for $\left(R_{A},R\right)\notin R_{\mathrm{AKW}}\left(p_{K},W\right)$. By Propositions 3 and 4, we obtain our main result shown below.

**Theorem** **4.** For any $R_{A},R>0$ and any $\left(p_{K},W\right)$, there exists a sequence of mappings ${\left\{\left(\varphi^{\left(n\right)},\psi^{\left(n\right)}\right)\right\}}_{n=1}^{\infty }$ such that for any $p_{X}\in P\left(X\right)$, we have

(22) $$\begin{array}{c} \frac{1}{n}-R\leq \frac{1}{n}\log|X^{m}|=\frac{m}{n}\log\left|X\right|\leq R,\\ p_{e}\left(\phi^{\left(n\right)},\psi^{\left(n\right)}|p_{X}^{n}\right)\leq e^{-n[E\left(R|p_{X}\right)-\delta_{1,n}]} \end{array}$$

and for any eavesdropper A with $\varphi_{A}$ satisfying $\varphi_{A}^{\left(n\right)}\in F_{A}^{\left(n\right)}\left(R_{A}\right)$, we have

(23) $$\begin{array}{c} \Delta^{\left(n\right)}\left(\varphi^{\left(n\right)},\varphi_{A}^{\left(n\right)}|p_{X}^{n},p_{K}^{n},W^{n}\right)\leq e^{-n[F\left(R_{A},R|p_{K},W\right)-\delta_{2,n}]}, \end{array}$$

where $\delta_{i,n},i=1,2$ are defined by

$$\begin{array}{c} \delta_{1,n}:=\frac{1}{n}\log\left[e{\left(n+1\right)}^{2|X|}\left\{{\left(n+1\right)}^{\left|X\right|}+1\right\}\right],\\ \delta_{2,n}:=\frac{1}{n}\log\left[5nR\{{\left(n+1\right)}^{\left|X\right|}+1\}\right]. \end{array}$$

Note that for i=1,2, $\delta_{i,n}\to 0$ as n→∞.

The functions $E(R|p_{X})$ and $F(R_{A},R|p_{K},W)$ take positive values if and only if $\left(R_{A},R\right)$ belongs to the set

$$\begin{array}{c} \left\{R>H\left(X\right)\right\}\cap R^{c}\left(p_{K},W\right):=R_{\mathrm{Sys}}^{\left(\mathrm{in}\right)}\left(p_{X},p_{K},W\right). \end{array}$$

Thus, by Theorem 4, under $\left(R_{A},R\right)\in R_{\mathrm{Sys}}^{\left(\mathrm{in}\right)}\left(p_{X},p_{K},W\right),$ we have the following:

- In terms of reliability, $p_{e}\left(\phi^{\left(n\right)},\psi^{\left(n\right)}|p_{X}^{n}\right)$ goes to zero exponentially as n tends to infinity, and its exponent is lower bounded by the function $E(R|p_{X})$.
- In terms of security, for any $\varphi_{A}$ satisfying $\varphi_{A}^{\left(n\right)}\in$$F_{A}^{\left(n\right)}\left(R_{A}\right)$, the information leakage $\Delta^{\left(n\right)}(\varphi^{\left(n\right)},\varphi_{A}^{\left(n\right)}$$|p_{X}^{n},p_{K}^{n},W^{n})$ on $X^{n}$ goes to zero exponentially as n tends to infinity, and its exponent is lower bounded by the function $F(R_{A},R|p_{K},W)$.
- The code that attains the exponent functions E($R|p_{X})$ is the universal code that depends only on R and not on the value of the distribution $p_{X}$.

Define

$$\begin{array}{c} D_{\mathrm{Sys}}^{\left(\mathrm{in}\right)}\left(p_{X},p_{K},W\right):=\left\{\left(R_{1},R_{2},E\left(R|p_{X}\right),F\left(R_{A},R|p_{K}\right)\right):\left(R_{1},R_{2}\right)\in R_{\mathrm{Sys}}^{\left(\mathrm{in}\right)}\left(p_{X},p_{K},W\right)\right\}. \end{array}$$

From Theorem 4, we immediately obtain the following corollary.

**Corollary** **1.** $$\begin{array}{c} R_{\mathrm{Sys}}^{\left(\mathrm{in}\right)}\left(p_{X},p_{K},W\right)\subseteq R_{\mathrm{Sys}}\left(p_{X},p_{K},W\right),D_{\mathrm{Sys}}^{\left(\mathrm{in}\right)}\left(p_{X},p_{K},W\right)\subseteq D_{\mathrm{Sys}}\left(p_{X},p_{K},W\right). \end{array}$$

**Figure 8:**
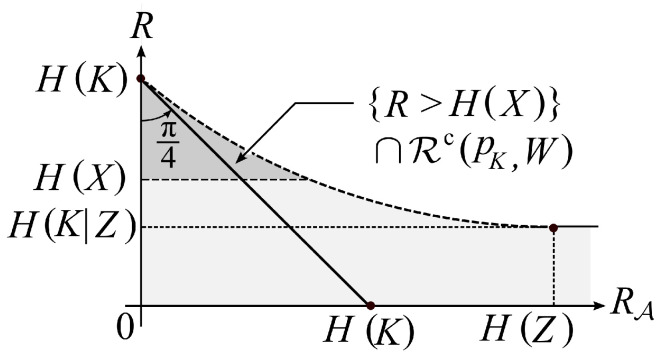
The inner bound $R_{\mathrm{Sys}}^{\left(\mathrm{in}\right)}\left(p_{X},p_{K},W\right)$ of the reliable and secure rate region $R_{\mathrm{Sys}}(p_{X},p_{K}$W).

A typical shape of $\left\{R>H\left(X\right)\right\}\cap R^{c}\left(p_{K},W\right)$ is shown in Figure 8.

## 6. Proofs of the Results

In this section, we prove our main theorem, i.e., Theorem 4.

### 6.1. Types of Sequences and Their Properties

In this subsection, we present basic results on the types. These results are basic tools for our analysis of several bounds related to the error provability of decoding or security.

**Definition** **5.** For any *n*-sequence $x^{n}=x_{1}x_{2}\cdots$ $x_{n}\in X^{n}$, $n(x|x^{n})$ denotes the number of *t* such that $x_{t}=x$. The relative frequency ${\left\{n(x|x^{n})/n\right\}}_{x\in X}$ of the components of $x^{n}$ is called the type of $x^{n}$ denoted by $P_{x^{n}}$. The set that consists of all the types on X is denoted by $P_{n}\left(X\right)$. Let $\bar{X}$ denote an arbitrary random variable whose distribution $P_{\bar{X}}$ belongs to $P_{n}\left(X\right)$. For $p_{\bar{X}}\in P_{n}\left(X\right)$, set $T_{\bar{X}}^{n}:=\left\{x^{n}:\;P_{x^{n}}=p_{\bar{X}}\right\}.$

For sets of types and joint types, the following lemma holds. For details of the proof, see Csiszár and Körner [21].

**Lemma** **4.**

(a) $\begin{array}{c} |P_{n}{\left(X\right)|\leq \left(n+1\right)}^{\left|X\right|}. \end{array}$
(b) For $P_{\bar{X}}\in P_{n}\left(X\right)$,
  $$\begin{array}{cc} {\left(n+1\right)}^{-\left|X\right|}e^{nH(\bar{X})} & \leq |T_{\bar{X}}^{n}|\leq e^{nH(\bar{X})}. \end{array}$$
(c) For $x^{n}\in T_{\bar{X}}^{n}$,
  $$\begin{array}{cc} p_{X}^{n}\left(x^{n}\right) & =e^{-n[H\left(\bar{X}\right)+D(p_{\bar{X}}\left|\right|p_{X})]}. \end{array}$$

By Lemma 4 parts (b) and (c), we immediately obtain the following lemma:

**Lemma** **5.** For $p_{\bar{X}}\in P_{n}\left(X\right)$,

$$\begin{array}{c} p_{X}^{n}\left(T_{\bar{X}}^{n}\right)\leq e^{-nD(p_{\bar{X}}||p_{X})}. \end{array}$$

### 6.2. Upper Bounds of $p_{\;e}\left(\phi^{\left(n\right)},\psi^{\left(n\right)}|p_{X}^{n}\right)$, and $\Delta_{n}(\varphi^{\left(n\right)},\varphi_{A}^{\left(n\right)}$$|p_{X}^{n},p_{K}^{n},W^{n})$

In this subsection, we evaluate upper bounds of $p_{e}($$\phi^{\left(n\right)},\psi^{\left(n\right)}\left|p_{X}^{n}\right)$ and $\Delta_{n}(\varphi^{\left(n\right)},\varphi_{A}^{\left(n\right)}$$|p_{X}^{n},p_{K}^{n},W^{n})$. For $p_{e}(\phi^{\left(n\right)}$$,\psi^{\left(n\right)}\left|p_{X}^{n}\right)$, we derive an upper bound that can be characterized with a quantity depending on $\left(\phi^{\left(n\right)},\psi^{\left(n\right)}\right)$ and type $P_{x^{n}}$ of sequences $x^{n}\in X^{n}$. We first evaluate $p_{e}\left(\phi^{\left(n\right)},\psi^{\left(n\right)}|p_{X}^{n}\right)$. For $x^{n}\in X^{n}$ and $p_{\bar{X}}\in P_{n}\left(X\right)$, we define the following functions:

$$\begin{array}{cc} \Xi_{x^{n}}\left(\phi^{\left(n\right)},\psi^{\left(n\right)}\right) & :=\left\{\begin{array}{ccc} 1 & \; & \mathrm{if}\;\psi^{\left(n\right)}\left(\phi^{\left(n\right)}\left(x^{n}\right)\right)\neq x^{n},\;\\ 0 & \; & \mathrm{otherwise}, \end{array}\right.\\ \Xi_{\bar{X}}\left(\phi^{\left(n\right)},\psi^{\left(n\right)}\right) & :=\frac{1}{|T_{\bar{X}}^{n}|}\sum_{x^{n}\in T_{\bar{X}}^{n}}\Xi_{x^{n}}\left(\phi^{\left(n\right)},\psi^{\left(n\right)}\right). \end{array}$$

Then we have the following lemma.

**Lemma** **6.** *In the proposed system, for any pair of* $(\phi^{\left(n\right)},$ $\psi^{\left(n\right)})$ *, we have*

(24) $$\begin{array}{c} p_{e}\left(\phi^{\left(n\right)},\psi^{\left(n\right)}|p_{X}^{n}\right)\leq \sum_{p_{\bar{X}}\in P_{n}\left(X\right)}\Xi_{\bar{X}}\left(\phi^{\left(n\right)},\psi^{\left(n\right)}\right)e^{-nD(p_{\bar{X}}||p_{X})}. \end{array}$$

**Proof.**  We have the following chain of inequalities:

$$\begin{array}{c} p_{e}\left(\phi^{\left(n\right)},\psi^{\left(n\right)}|p_{X}^{n}\right)\overset{\left(a\right)}{=}\sum_{p_{\bar{X}}\in P_{n}\left(X\right)}\sum_{x^{n}\in T_{\bar{X}}^{n}}\Xi_{x^{n}}\left(\phi^{\left(n\right)},\psi^{\left(n\right)}\right)p_{X}^{n}\left(x^{n}\right)\\ =\sum_{p_{\bar{X}}\in P_{n}\left(X\right)}\frac{1}{|T_{\bar{X}}^{n}|}\sum_{x^{n}\in T_{\bar{X}}^{n}}\Xi_{x^{n}}\left(\phi^{\left(n\right)},\psi^{\left(n\right)}\right)\left|T_{\bar{X}}^{n}\right|p_{X}^{n}\left(x^{n}\right)\\ \overset{\left(b\right)}{=}\sum_{p_{\bar{X}}\in P_{n}\left(X\right)}\frac{1}{|T_{\bar{X}}^{n}|}\sum_{x^{n}\in T_{\bar{X}}^{n}}\Xi_{x^{n}}\left(\phi^{\left(n\right)},\psi^{\left(n\right)}\right)p_{X}^{n}\left(T_{\bar{X}}^{n}\right)\overset{\left(c\right)}{=}\sum_{p_{\bar{X}}\in P_{n}\left(X\right)}\Xi_{\bar{X}}\left(\phi^{\left(n\right)},\psi^{\left(n\right)}\right)p_{X}^{n}\left(T_{\bar{X}}^{n}\right)\\ \overset{\left(d\right)}{\leq }\sum_{p_{\bar{X}}\in P_{n}\left(X\right)}\Xi_{\bar{X}}\left(\phi^{\left(n\right)},\psi^{\left(n\right)}\right)e^{-nD(p_{\bar{X}}||p_{X})}. \end{array}$$

Step (a) follows from the definition of $\Xi_{x^{n}}\left(\phi^{\left(n\right)},\psi^{\left(n\right)}\right)$. Step (b) follows from the probabilities $p_{X}^{n}\left(x^{n}\right)$ for $x^{n}\in T_{\bar{X}}^{n}$ taking an identical value. Step (c) follows from the definition of $\Xi_{\bar{X}}\left(\phi^{\left(n\right)},\psi^{\left(n\right)}\right)$. Step (d) follows from Lemma 5. □

### 6.3. Random Coding Arguments

We construct a pair of affine encoders $\varphi^{\left(n\right)}=\left(\varphi_{1}^{\left(n\right)},\varphi_{e}^{\left(n\right)}\right)$ using the random coding method. For the joint decoder $\psi^{\left(n\right)}$, we propose the minimum entropy decoder used in Csiszár [6] and Oohama and Han [22].

*Random Construction of Affine Encoders:* We first choose *m* such that

$$m:=\left\lfloor \frac{nR}{\log|X|}\right\rfloor ,$$

where ⌊a⌋ stands for the integer part of a. It is obvious that

$$R-\frac{1}{n}\leq \frac{m}{n}\log\left|X\right|\leq R.$$

By definition (2) of $\phi^{\left(n\right)}$, we have that for $x^{n}\in X^{n}$,

$$\begin{array}{c} \phi^{\left(n\right)}\left(x^{n}\right)=x^{n}A, \end{array}$$

where *A* is a matrix with *n* rows and *m* columns. By definition (3) of $\varphi^{\left(n\right)}$, we have that for $k^{n}\in X^{n}$,

$$\begin{array}{c} \varphi^{\left(n\right)}\left(k^{n}\right)=k^{n}A+b^{m}, \end{array}$$

where $b^{m}$ is a vector with m columns. Entries of *A* and $b^{m}$ are from the field of X. These entries are selected at random, independently of each other, and with a uniform distribution. Randomly constructed linear encoder $\phi^{\left(n\right)}$ and affine encoder $\varphi^{\left(n\right)}$ have three properties shown in the following lemma.

**Lemma** **7** (Properties of Linear/Affine Encoders)**.**

*(a)*  *For any $x^{n},v^{n}\in X^{n}$ with $x^{n}\neq v^{n}$, we have*
  (25) $$\begin{array}{c} \mathrm{Pr}\left[\phi^{\left(n\right)}\left(x^{n}\right)=\phi^{\left(n\right)}\left(v^{n}\right)\right]=\mathrm{Pr}\left[\left(x^{n}⊖v^{n}\right)A=0^{m}\right]={\left|X\right|}^{-m}. \end{array}$$
*(b)*  *For any $s^{n}\in X^{n}$ and for any ${\tilde{s}}^{m}\in X^{m}$, we have*
  (26) $$\begin{array}{c} \mathrm{Pr}\left[\varphi^{\left(n\right)}\left(s^{n}\right)={\tilde{s}}^{m}\right]=\mathrm{Pr}\left[s^{n}A⊕b^{m}={\tilde{s}}^{m}\right]={\left|X\right|}^{-m}. \end{array}$$
*(c)*  *For any $s^{n},t^{n}\in X^{n}$ with $s^{n}\neq t^{n}$, and for any ${\tilde{s}}^{m}\in X^{m}$, we have*
  (27) $$\begin{array}{c} \mathrm{Pr}\left[\varphi^{\left(n\right)}\left(s^{n}\right)=\varphi^{\left(n\right)}\left(t^{n}\right)={\tilde{s}}^{m}\right]=\mathrm{Pr}\left[s^{n}A⊕b^{m}=t^{n}A⊕b^{m}={\tilde{s}}^{m}\right]={\left|X\right|}^{-2m}. \end{array}$$

Proof of this lemma is given in Appendix B. We next define the decoder function $\psi^{\left(n\right)}:X^{m}\to X^{n}.$ To this end, we define the following quantities.

**Definition** **6.** For $x^{n}\in X^{n}$, we denote the entropy calculated from the type $P_{x^{n}}$ by $H(x^{n})$. In other words, for a type $P_{\bar{X}}\in P_{n}\left(X\right)$ such that $P_{\bar{X}}=P_{x^{n}}$, we define $H\left(x^{n}\right)=H\left(\bar{X}\right)$.

*Minimum Entropy Decoder:* For $\phi^{\left(n\right)}\left(x^{n}\right)={\tilde{x}}^{m}$, we define the decoder function $\psi^{\left(n\right)}:X^{m}\to X^{n}$ as follows:

$$\psi^{\left(n\right)}\left({\tilde{x}}^{m}\right):=\left\{\begin{array}{cc} {\hat{x}}^{n} & \mathrm{if}\;\phi^{\left(n\right)}\left({\hat{x}}^{n}\right)={\tilde{x}}^{m},\\ & \mathrm{and}\;H\left({\hat{x}}^{n}\right)<H\left({\overset{ˇ}{x}}^{n}\right)\\ & \mathrm{for}\;\mathrm{all}\;{\overset{ˇ}{x}}^{n}\mathrm{such}\;\mathrm{that}\;\\ & \;\phi^{\left(n\right)}\left({\overset{ˇ}{x}}^{n}\right)={\tilde{x}}^{m},\\ & \mathrm{and}\;{\overset{ˇ}{x}}^{n}\neq {\hat{x}}^{n},\;\\ \mathrm{arbitrary} & \mathrm{if}\;\mathrm{there}\;\mathrm{is}\;\mathrm{no}\;\mathrm{such}\;{\hat{x}}^{n}\in X^{n}. \end{array}\right.$$

*Error Probability Bound:* In the following arguments, we let expectations based on the random choice of the affine encoder $\varphi^{\left(n\right)}$ be denoted by E[·]. Define

$$\Lambda_{\bar{X}}\left(R\right):=e^{-n{\left[R-H\left(\bar{X}\right)\right]}^{+}}.$$

Then we have the following lemma.

**Lemma** **8.** *For any n and for any*$P_{\bar{X}}\in P_{n}\left(X\right)$,

$$E\left[\Xi_{\bar{X}}\left(\phi^{\left(n\right)},\psi^{\left(n\right)}\right)\right]\leq e{\left(n+1\right)}^{\left|X\right|}\Lambda_{\bar{X}}\left(R\right).$$

Proof of this lemma is given in Appendix C.

*Estimation of Approximation Error:* Define

$$\begin{array}{c} \Theta \left(R,\varphi_{A}^{\left(n\right)}|p_{K^{n}},W^{n}\right):=\sum_{\left(a,k^{n}\right)\in M_{A}^{\left(n\right)}\times X^{n}}p_{M_{A}^{\left(n\right)}K^{n}}\left(a,k^{n}\right)\log\left[1+\left(e^{nR}-1\right)p_{K^{n}|M_{A}^{\left(n\right)}}\left(k^{n}|a\right)\right]. \end{array}$$

Then we have the following lemma.

**Lemma** **9.** *For any*n,m*satisfying*(m/n)log|X|≤R, *we have*

(28) $$\begin{array}{c} E\left[D\;\;\left.\left.\left.\left(p_{{\tilde{K}}^{m}|M_{A}^{\left(n\right)}}\right|\right|p_{V^{m}}\right|p_{M_{A}^{\left(n\right)}}\right)\right]\leq \Theta \left(R,\varphi_{A}^{\left(n\right)}|p_{K^{n}},W^{n}\right). \end{array}$$

Proof of this lemma is given in Appendix D. From the bound (28) in Lemma (9), we know that the quantity $\Theta (R,\varphi_{A}^{\left(n\right)}|p_{K^{n}},W^{n})$ serves as an upper bound of the ensemble average of the conditional divergence $D(p_{{\tilde{K}}^{m}|M_{A}^{\left(n\right)}}$$\left|\right|p_{V^{m}}\left|p_{M_{A}^{\left(n\right)}}\right).$ Hayashi [23] obtained the same upper bound of the ensemble average of the conditional divergence for an ensemble of universal${}_{2}$ functions. In this paper, we prove the bound (28) for an ensemble of affine encoders. To derive this bound, we need to use Lemma 7 parts (b) and (c), the two important properties that a class of random affine encoders satisfies. From Lemmas 1 and 9, we have the following corollary.

**Corollary** **2.** $$\begin{array}{c} E\left[\Delta_{n}\left(\varphi^{\left(n\right)},\varphi_{A}^{\left(n\right)}|p_{X}^{n},p_{K}^{n},W^{n}\right)\right]\leq \Theta \left(R,\varphi_{A}^{\left(n\right)}|p_{K}^{n},W^{n}\right). \end{array}$$

*Existence of Good Universal Code $\left(\varphi^{\left(n\right)},\psi^{\left(n\right)}\right)$:*

From Lemma 8 and Corollary 2, we have the following lemma stating the existence of a good universal code $\left(\varphi^{\left(n\right)},\psi^{\left(n\right)}\right)$.

**Lemma** **10.** *There exists at least one deterministic code*$\left(\varphi^{\left(n\right)},\psi^{\left(n\right)}\right)$*satisfying*(m/n)log|X|≤R, *such that for any*$p_{\bar{X}}$$\in P_{n}\left(X\right)$,

$$\begin{array}{c} \Xi_{\bar{X}}\left(\phi^{\left(n\right)},\psi^{\left(n\right)}\right)\leq e{\left(n+1\right)}^{\left|X\right|}\left\{{\left(n+1\right)}^{\left|X\right|}+1\right\}\Lambda_{\bar{X}}\left(R\right). \end{array}$$

*Furthermore, for any*$\varphi_{A}^{\left(n\right)}\in F_{A}^{\left(n\right)}\left(R_{A}\right)$, *we have*

$$\begin{array}{c} \Delta_{n}\left(\varphi^{\left(n\right)},\varphi_{A}^{\left(n\right)}|p_{X}^{n},p_{K}^{n},W^{n}\right)\leq \left\{{\left(n+1\right)}^{\left|X\right|}+1\right\}\Theta \left(R,\varphi_{A}^{\left(n\right)}|p_{K}^{n},W^{n}\right). \end{array}$$

**Proof.**  We have the following chain of inequalities:

$$\begin{array}{c} E\left[\sum_{p_{\bar{X}}\in P_{n}\left(X\right)}\frac{\Xi_{\bar{X}}\left(\phi^{\left(n\right)},\psi^{\left(n\right)}\right)}{e{\left(n+1\right)}^{\left|X\right|}\Lambda_{\bar{X}}\left(R\right)}+\frac{\Delta_{n}\left(\varphi^{\left(n\right)},\varphi_{A}^{\left(n\right)}|p_{X}^{n},p_{K}^{n},W^{n}\right)}{\Theta (R,\varphi_{A}^{\left(n\right)}|p_{K}^{n},W^{n})}\right]\\ =\sum_{p_{\bar{X}}\in P_{n}\left(X\right)}\frac{E\left[\Xi_{\bar{X}}\left(\phi^{\left(n\right)},\psi^{\left(n\right)}\right)\right]}{e{\left(n+1\right)}^{\left|X\right|}\Lambda_{\bar{X}}\left(R\right)}+\frac{E\left[\Delta_{n}\left(\varphi^{\left(n\right)},\varphi_{A}^{\left(n\right)}|p_{X}^{n},p_{K}^{n},W^{n}\right)\right]}{\Theta (R,\varphi_{A}^{\left(n\right)}|p_{K}^{n},W^{n})}\\ \overset{\left(a\right)}{\leq }\sum_{p_{\bar{X}}\in P_{n}\left(X\right)}1+1=\left|P_{n}\left(X\right)\right|+1\overset{\left(b\right)}{\leq }{\left(n+1\right)}^{\left|X\right|}+1. \end{array}$$

Step (a) follows from Lemma 8 and Corollary 2. Step (b) follows from Lemma 4 part (a). Hence, there exists at least one deterministic code $\left(\varphi^{\left(n\right)},\psi^{\left(n\right)}\right)$ such that

$$\begin{array}{c} \sum_{p_{\bar{X}}\in P_{n}\left(X\right)}\frac{\Xi_{\bar{X}}\left(\phi^{\left(n\right)},\psi^{\left(n\right)}\right)}{e{\left(n+1\right)}^{\left|X\right|}\Lambda_{\bar{X}}\left(R\right)}+\frac{\Delta_{n}\left(\varphi^{\left(n\right)},\varphi_{A}^{\left(n\right)}|p_{X}^{n},p_{K}^{n},W^{n}\right)}{\Theta (R,\varphi_{A}^{\left(n\right)}|p_{K}^{n},W^{n})}\leq {\left(n+1\right)}^{\left|X\right|}+1, \end{array}$$

from which we have that

$$\begin{array}{c} \frac{\Xi_{\bar{X}}\left(\phi^{\left(n\right)},\psi^{\left(n\right)}\right)}{e{\left(n+1\right)}^{\left|X\right|}\Lambda_{\bar{X}}\left(R\right)}\leq {\left(n+1\right)}^{\left|X\right|}+1, \end{array}$$

for any $p_{\bar{X}}\in P_{n}\left(X\right)$. Furthermore, we have that for any $\varphi_{A}^{\left(n\right)}\in F_{A}^{\left(n\right)}\left(R_{A}\right)$,

$$\begin{array}{c} \frac{\Delta_{n}\left(\varphi^{\left(n\right)},\varphi_{A}^{\left(n\right)}|p_{X}^{n},p_{K}^{n},W^{n}\right)}{\Theta (R,\varphi_{A}^{\left(n\right)}|p_{K}^{n},W^{n})}\leq {\left(n+1\right)}^{\left|X\right|}+1, \end{array}$$

completing the proof. □

**Proposition** **5.** *For any*$R_{A},R>0$*and any*$\left(p_{K},W\right)$, *there exists a sequence of mappings*${\left\{\left(\varphi^{\left(n\right)},\psi^{\left(n\right)}\right)\right\}}_{n=1}^{\infty }$*such that for any*$p_{X}\in P\left(X\right)$, *we have*

(29) $$\begin{array}{c} R-\frac{1}{n}\leq \frac{1}{n}\log|X^{m}|=\frac{m}{n}\log\left|X\right|\leq R,\\ p_{e}\left(\phi^{\left(n\right)},\psi^{\left(n\right)}|p_{X}^{n}\right)\leq e{\left(n+1\right)}^{2|X|}\{{\left(n+1\right)}^{\left|X\right|}+1\}e^{-n[E\left(R|p_{X}\right)]} \end{array}$$

*and for any eavesdropper*A*with*$\varphi_{A}$*satisfying*$\varphi_{A}^{\left(n\right)}\in F_{A}^{\left(n\right)}\left(R_{A}\right)$, *we have*

(30) $$\begin{array}{c} \Delta^{\left(n\right)}\left(\varphi^{\left(n\right)},\varphi_{A}^{\left(n\right)}|p_{X}^{n},p_{K}^{n},W^{n}\right)\leq \left\{{\left(n+1\right)}^{\left|X\right|}+1\right\}\Theta \left(R,\varphi_{A}^{\left(n\right)}|p_{K}^{n},W^{n}\right). \end{array}$$

**Proof.**  By Lemma 10, there exists $(\varphi^{\left(n\right)},$$\psi^{\left(n\right)})$ satisfying (m/n)log|X|≤R such that for any $p_{\bar{X}}$ $\in P_{n}\left(X\right)$,

(31) $$\begin{array}{c} \Xi_{\bar{X}}\left(\phi^{\left(n\right)},\psi^{\left(n\right)}\right)\leq e{\left(n+1\right)}^{\left|X\right|}\left\{{\left(n+1\right)}^{\left|X\right|}+1\right\}\Lambda_{\bar{X}}\left(R\right). \end{array}$$

Furthermore, for any $\varphi_{A}^{\left(n\right)}\in F_{A}^{\left(n\right)}\left(R_{A}\right)$,

(32) $$\begin{array}{c} \Delta_{n}\left(\varphi^{\left(n\right)},\varphi_{A}^{\left(n\right)}|p_{X}^{n},p_{K}^{n},W^{n}\right)\leq \left\{{\left(n+1\right)}^{\left|X\right|}+1\right\}\Theta \left(R,\varphi_{A}^{\left(n\right)}|p_{K}^{n},W^{n}\right). \end{array}$$

The bound (30) in Proposition 5 has already been proven in (32). Hence, it suffices to prove the bound (29) in Proposition 5 to complete the proof. On an upper bound of $p_{e}\left(\phi^{\left(n\right)},\psi^{\left(n\right)}|p_{X}^{n}\right)$, we have the following chain of inequalities:

$$\begin{array}{l} p_{e}\left(\phi^{\left(n\right)},\psi^{\left(n\right)}|p_{X}^{n}\right)\overset{\left(a\right)}{\leq }e{\left(n+1\right)}^{\left|X\right|}\left\{{\left(n+1\right)}^{\left|X\right|}+1\right\}\sum_{p_{\bar{X}}\in P_{n}\left(X\right)}\Lambda_{\bar{X}}\left(R\right)e^{-nD(p_{\bar{X}}||p_{X})}\\ \leq e{\left(n+1\right)}^{\left|X\right|}\left\{{\left(n+1\right)}^{\left|X\right|}+1\right\}\left|P_{n}\left(X\right)\right|e^{-n[E\left(R|p_{X}\right)]}\overset{\left(c\right)}{\leq }e{\left(n+1\right)}^{2|X|}\left\{{\left(n+1\right)}^{\left|X\right|}+1\right\}e^{-nE(R|p_{X})}. \end{array}$$

Step (a) follows from Lemma 6 and (31). Step (b) follows from Lemma 4 part (a). □

### 6.4. Explicit Upper Bound of $\Theta (R_{1},R_{2},\varphi_{A}^{\left(n\right)}|p_{ZK_{1}K_{2}}^{n})$

In this subsection, we derive an explicit upper bound of $\Theta (R,\varphi_{A}^{\left(n\right)}|p_{K}^{n},W^{n})$ that holds for any eavesdropper A with $\varphi_{A}$ satisfying $\varphi_{A}^{\left(n\right)}\in F_{A}^{\left(n\right)}\left(R_{A}\right)$. Here we recall the following definitions:

$$\begin{array}{c} {℘}_{\eta }^{\left(n\right)}={℘}_{\eta }^{\left(n\right)}\left(R|p_{K}^{n},W^{n}\right):=p_{M_{A}^{\left(n\right)}Z^{n}K^{n}}\{R\geq \left.\frac{1}{n}\log\frac{1}{p_{K^{n}|M_{A}^{\left(n\right)}}\left(K^{n}|M_{A}^{\left(n\right)}\right)}-\eta \right\},\\ \Phi_{D,\eta }^{\left(n\right)}\left(R_{A},R|p_{K}^{n}W^{n}\right):=\max_{\varphi_{A}^{\left(n\right)}\in F^{\left(n\right)}\left(R_{A}\right)}\left\{nR{℘}_{\eta }^{\left(n\right)}\left(R|p_{K}^{n},W^{n}\right)+e^{-n\eta }\right\},\\ \Phi_{D}^{\left(n\right)}\left(R_{A},R|p_{K}^{n}W^{n}\right):=\underset{\eta >0}{\inf}\Phi_{D,\eta }^{\left(n\right)}\left(R_{A},R|p_{K}^{n}W^{n}\right). \end{array}$$

Then we have the following lemma.

**Lemma** **11.** *For any*η>0*and for any eavesdropper*A*with*$\varphi_{A}$*satisfying*$\varphi_{A}^{\left(n\right)}\in F_{A}^{\left(n\right)}\left(R_{A}\right)$, *we have*

(33) $$\begin{array}{c} \Theta \left(R,\varphi_{A}^{\left(n\right)}|p_{K}^{n},W^{n}\right)\leq \Phi_{D,\eta }^{\left(n\right)}\left(R_{A},R|p_{K}^{n}W^{n}\right), \end{array}$$

*which implies that*

(34) $$\begin{array}{c} \Theta \left(R,\varphi_{A}^{\left(n\right)}|p_{K}^{n},W^{n}\right)\leq \Phi_{D}^{\left(n\right)}\left(R_{A},R|p_{K}^{n}W^{n}\right). \end{array}$$

**Proof.**  We first observe that

(35) $$\begin{array}{c} \Theta \left(R,\varphi_{A}^{\left(n\right)}|p_{K}^{n},W^{n}\right)=E\left[\log\left\{1+\left(e^{nR}-1\right)p_{K^{n}|M_{A}^{\left(n\right)}}\left(K^{n}|M_{A}^{\left(n\right)}\right)\right\}\right]. \end{array}$$

We further observe the following:

(36) $$\begin{array}{c} R<\frac{1}{n}\log\frac{1}{p_{K^{n}|M_{A}^{\left(n\right)}}\left(K^{n}|M_{A}^{\left(n\right)}\right)}-\eta \Leftrightarrow e^{nR}p_{K^{n}|M_{A}^{\left(n\right)}}\left(K^{n}|M_{A}^{\left(n\right)}\right)<e^{-n\eta }\\ \Rightarrow \log\left\{1+e^{nR}p_{K^{n}|M_{A}^{\left(n\right)}}\left(K^{n}|M_{A}^{\left(n\right)}\right)\right\}\leq \log\left(1+e^{-n\eta }\right)\\ \overset{\left(a\right)}{\Rightarrow }\log\left\{1+e^{nR}p_{K^{n}|M_{A}^{\left(n\right)}}\left(K^{n}|M_{A}^{\left(n\right)}\right)\right\}\leq e^{-n\eta }\\ \Rightarrow \log\left\{1+\left(e^{nR}-1\right)p_{K^{n}|M_{A}^{\left(n\right)}}\left(K^{n}|M_{A}^{\left(n\right)}\right)\right\}\leq e^{-n\eta }. \end{array}$$

Step (a) follows from log(1+a)≤a. We also note that

(37) $$\begin{array}{c} \log\left\{1+\left(e^{nR}-1\right)p_{K^{n}|M_{A}^{\left(n\right)}}\left(K^{n}|M_{A}^{\left(n\right)}\right)\right\}\leq \log\left[e^{nR}\right]=nR. \end{array}$$

From (35), (36), and (37) we have the bound (33) in Lemma 11. □

**Proof** **of** **Proposition** **3:** This proposition immediately follows from Proposition 5 and Lemma 11. □

For the upper bound of ${℘}_{\eta }^{\left(n\right)}$, we have the following lemma.

**Lemma** **12.** *For any*η>0*and for any eavesdropper*A*with*$\varphi_{A}$*satisfying*$\varphi_{A}^{\left(n\right)}\in F_{A}^{\left(n\right)}\left(R_{A}\right)$, *we have*${℘}_{\eta }^{\left(n\right)}\leq {\tilde{℘}}_{\eta }^{\left(n\right)}+3e^{-n\eta }$*, where*

$$\begin{array}{c} {\tilde{℘}}_{\eta }^{\left(n\right)}:=p_{M_{A}^{\left(n\right)}Z^{n}K^{n}}\{ \end{array}$$

(38) $$\begin{array}{cc} 0 & \geq \frac{1}{n}\log\frac{{\hat{q}}_{M_{A}^{\left(n\right)}Z^{n}K^{n}}\left(M_{A}^{\left(n\right)},Z^{n},K^{n}\right)}{p_{M_{A}^{\left(n\right)}Z^{n}K^{n}}\left(M_{A}^{\left(n\right)},Z^{n},K^{n}\right)}-\eta , \end{array}$$

(39) $$\begin{array}{cc} 0 & \geq \frac{1}{n}\log\frac{q_{Z^{n}}\left(Z^{n}\right)}{p_{Z^{n}}\left(Z^{n}\right)}-\eta , \end{array}$$

$$\begin{array}{cc} R_{A} & \geq \frac{1}{n}\log\frac{p_{Z^{n}|M_{A}^{\left(n\right)}}\left(Z^{n}|M_{A}^{\left(n\right)}\right)}{p_{Z^{n}}\left(Z^{n}\right)}-\eta , \end{array}$$

(40) $$\begin{array}{cc} R & \geq \frac{1}{n}\log\frac{1}{p_{K^{n}|M_{A}^{\left(n\right)}}\left(K^{n}|M_{A}^{\left(n\right)}\right)}-\eta \}. \end{array}$$

*The probability distributions appearing in the two inequalities (38) and (39) in the right members of (40) have a property that we can select them arbitrarily. In (38), we can choose any probability distribution*${\hat{q}}_{M_{A}^{\left(n\right)}Z^{n}K^{n}}$*on*$M_{A}^{\left(n\right)}$$\times Z^{n}$$\times X^{n}$*. In (39), we can choose any distribution*$q_{Z^{n}}$*on*$Z^{n}$.

Proof of this lemma is given in Appendix E.

**Proof** **of** **Proposition** **4:** The claim of Proposition 4 is that for n≥1/R,

(41) $$\begin{array}{c} \Phi_{D}^{\left(n\right)}\left(R_{A},R|p_{K}^{n}W^{n}\right)\leq 5nRe^{-nF(R_{A},R|p_{K},W)}. \end{array}$$

By Lemma 12 and the definition of $\Phi_{D,\eta }^{\left(n\right)}\left(R_{A},R|p_{K}^{n}W^{n}\right)$, we have that for n≥1/R,

(42) $$\begin{array}{c} \Phi_{D,\eta }^{\left(n\right)}\left(R_{A},R|p_{K}^{n}W^{n}\right)\leq nR\left({\tilde{℘}}_{\eta }^{\left(n\right)}+4e^{-n\eta }\right). \end{array}$$

The quantity ${\tilde{℘}}_{\eta }^{\left(n\right)}+4e^{-n\eta }$ is the same as the upper bound on the correct probability of decoding for one helper source coding problem in Lemma 1 in Oohama [9] (extended version). In a manner similar to the derivation of the exponential upper bound of the correct probability of decoding for one helper source coding problem, we can prove that for any $\varphi_{A}^{\left(n\right)}\in F_{A}^{\left(n\right)}\left(R_{A}\right)$ and for some $\eta^{*}=\eta^{*}\left(n,R_{A},R\right)$, we have

(43) $$\begin{array}{c} {\tilde{℘}}_{\eta^{*}}^{\left(n\right)}+4e^{-n\eta^{*}}\leq 5e^{-nF(R_{A},R|p_{K},W)}. \end{array}$$

From (42), (43), and the definition of $\Phi_{D}^{\left(n\right)}\left(R_{A},R|p_{K}^{n}W^{n}\right)$, we have (41). □

## 7. Conclusions

In this paper, we have proposed a novel security model for analyzing the security of Shannon cipher systems against an adversary that is not only eavesdropping the public communication channel to obtain ciphertexts but is also obtaining some physical information leaked by the device implementing the cipher system through side-channel attacks. We have also presented a countermeasure against such an adversary in the case of one-time pad encryption by using an affine encoder with certain properties. The main distinguishing feature of our countermeasure is that it is independent of the characteristics or the types of physical information leaked from the devices on which the cipher system is implemented.

## Figures and Tables

**Table 1:** Differences between Problems 1, 2, and 3 in terms of ${\left\{\varphi^{\left(n\right)}\right\}}_{n\geq 1}$ and security criteria.

	Problem 1	Problem 2	Problem 3
$\varphi^{\left(n\right)}$	affine encoders	general	general
Security Criteria	$D(p_{{\tilde{K}}^{m}|M_{A}^{\left(n\right)}}||p_{V^{m}}|p_{M_{A}^{\left(n\right)}})$	$d(p_{V^{m}}\times p_{M_{A}^{\left(n\right)}},p_{{\tilde{K}}^{m}M_{A}^{\left(n\right)}})$	$p_{c,A}^{\left(n\right)}\left(\varphi^{\left(n\right)},\varphi_{A}^{\left(n\right)},\psi_{A}^{\left(n\right)}|p_{K}^{n},W^{n}\right)$

## Appendix A. Correct Probability of Decoding and Variational Distance

In this appendix, we prove Lemma 3.

For $a\in M_{A}^{\left(n\right)}$, we set

$$D\left(a\right)=\left\{{\tilde{k}}^{m}:{\tilde{k}}^{m}=\varphi^{\left(n\right)}\left(k^{n}\right)\;\mathrm{and}\;\psi_{A}^{\left(n\right)}\left({\tilde{k}}^{m},a\right)=k^{n}\;\mathrm{for}\;\mathrm{some}\;k^{n}\in X^{n}\right\}.$$

Then we have the following chain of inequalities:

$$\begin{array}{c} d\left(p_{V^{m}}\times p_{M_{A}^{\left(n\right)}},p_{{\tilde{K}}^{m}M_{A}^{\left(n\right)}}\right)=\sum_{a\in M_{A}^{\left(n\right)}}p_{M_{A}^{\left(n\right)}}\left(a\right)\sum_{{\tilde{k}}^{m}\in X^{m}}\left|p_{{\tilde{K}}^{m}|M_{A}^{\left(n\right)}}\left({\tilde{k}}^{m}|a\right)-\frac{1}{{\left|X\right|}^{m}}\right|\\ \geq \sum_{a\in M_{A}^{\left(n\right)}}p_{M_{A}^{\left(n\right)}}\left(a\right)\left\{p_{{\tilde{K}}^{m}|M_{A}^{\left(n\right)}}\left(D(a)|a\right)-\frac{\left|D(a)\right|}{{\left|X\right|}^{m}}\right\}=\sum_{a\in M_{A}^{\left(n\right)}}p_{M_{A}^{\left(n\right)}}\left(a\right)\left\{p_{{\tilde{K}}^{m}|M_{A}^{\left(n\right)}}\left(D(a)|a\right)-\frac{1}{{\left|X\right|}^{m}}\right\}\\ =p_{c,A}^{\left(n\right)}\left(\varphi^{\left(n\right)},\varphi_{A}^{\left(n\right)},\psi_{A}^{\left(n\right)}|p_{K}^{n},W^{n}\right)-\frac{1}{{\left|X\right|}^{m}}, \end{array}$$

completing the proof. □

## Appendix B. Proof of Lemma 7

Let $a_{l}^{m}$ be the l-th low vector of the matrix *A*. For each l=1,2,⋯,n, let $A_{l}^{m}\in X^{m}$ be a random vector that represents the randomness of the choice of $a_{l}^{m}\in X^{m}$. Let $B^{m}\in X^{m}$ be a random vector that represents the randomness of the choice of $b^{m}\in X^{m}$. We first prove part (a). Without loss of generality, we may assume $x_{1}\neq v_{1}$. Under this assumption, we have the following:

(A1) $$\begin{array}{c} \left(x^{n}⊖v^{n}\right)A=0^{m}\Leftrightarrow \sum_{l=1}^{n}\left(x_{l}⊖v_{l}\right)a_{l}^{m}=0^{m}\Leftrightarrow a_{1}^{m}=\sum_{l=2}^{n}\frac{v_{l}⊖x_{l}}{x_{1}⊖v_{1}}a_{l}^{m}. \end{array}$$

Computing $\mathrm{Pr}[\phi \left(x^{n}\right)=\phi \left(v^{n}\right)]$, we have the following chain of equalities:

Pr[ϕ(xn)=ϕ(vn)]=Pr[(yn⊖wn)A=0m]=(a)Pra1m=∑l=2nwl⊖ylx1⊖v1alm=(b)∑alml=2n∈X(n−1)m∏l=2nPAlm(alm)PA1m∑l=2nwl⊖xly1⊖v1alm=|X|−m∑alml=2n∈X(n−1)m∏l=2nPAlm(alm)=|X|−m.

Step (a) follows from (A1). Step (b) follows from that n random vectors $A_{l}^{m},l=1,2,\cdots ,n$ are independent. We next prove part b. We have the following:

(A2) $$\begin{array}{c} s^{n}A⊕b^{m}={\tilde{s}}^{m}\Leftrightarrow b^{m}={\tilde{s}}^{m}⊖\left\{\sum_{l=1}^{n}s_{l}a_{l}^{m}\right\}. \end{array}$$

Computing $\mathrm{Pr}[s^{n}A⊕b^{m}={\tilde{s}}^{m}]$, we have the following chain of equalities:

Pr[snA⊕bm=s˜m]=(a)Prbm=s˜m⊖∑l=1nslalm=(b)∑alml=1n∈Xnm∏l=1nPAlm(alm)PBms˜m⊖∑l=1nslalm=(c)|X|−m∑alml=1n∈Xnm∏l=1nPAlm(alm)=|X|−m.

Step (a) follows from (A2). Step (b) follows from that n random vectors $A_{l}^{m},l=1,2,\cdots ,n$ and $B^{m}$ are independent. We finally prove the part (c). We first observe that $s^{n}\neq t^{n}\Leftrightarrow$ is equivalent to $s_{i}\neq t_{i}\;\mathrm{for}\;\mathrm{some}\;i\in \left\{1,2,\cdots ,n\right\}.$ Without loss of generality, we may assume that $s_{1}\neq t_{1}$. Under this assumption, we have the following:

(A3) $$\begin{array}{c} \begin{array}{c} s^{n}A⊕b^{m}=t^{n}A⊕b^{m}={\tilde{s}}^{m} \end{array}\\ \Leftrightarrow \begin{array}{c} \left(s^{n}⊖t^{n}\right)A=0,b^{m}={\tilde{s}}^{m}⊖\left\{\sum_{l=1}^{n}s_{l}a_{l}^{m}\right\} \end{array}\\ \Leftrightarrow \begin{array}{c} a_{1}^{m}=\sum_{l=2}^{n}\frac{t_{l}⊖s_{l}}{s_{1}⊖t_{1}}a_{l}^{m},b^{m}={\tilde{s}}^{m}⊖\left\{\sum_{l=1}^{n}s_{l}a_{l}^{m}\right\} \end{array}\\ \Leftrightarrow \begin{array}{c} a_{1}^{m}=\sum_{l=2}^{n}\frac{t_{l}⊖s_{l}}{s_{1}⊖t_{1}}a_{l}^{m},b^{m}={\tilde{s}}^{m}⊕\sum_{l=2}^{n}\frac{t_{1}s_{l}⊖s_{1}t_{l}}{s_{1}⊖t_{1}}a_{l}^{m}. \end{array} \end{array}$$

Computing $\mathrm{Pr}[s^{n}A⊕b^{m}=t^{n}A⊕b^{m}={\tilde{s}}^{m}]$, we have the following chain of equalities:

Pr[snA⊕bm=tnA⊕bm=s˜m]=(a)Pra1m=∑l=2ntl⊖sls1⊖t1alm∧bm=s˜m⊕∑l=2nt1sl⊖s1tls1⊖t1alm=(b)∑alml=2n∈X(n−1)m∏l=2nPAlm(alm)PA1m∑l=2ntl⊖sls1⊖t1almPBms˜m⊕∑l=2nt1sl⊖s1tls1⊖t1alm=|X|−2m∑alml=2n∈X(n−1)m∏l=2nPAlm(alm)=|X|−2m.

Step (a) follows from (A3). Step (b) follows from the independent property on $A_{l}^{m},l=1,2,\cdots ,n$ and $B^{m}.$ □

## Appendix C. Proof of Lemma 8

In this appendix, we provide the proof of Lemma 8.

For simplicity of notation, we write $M={\left|X\right|}^{m}$. For $x^{n}\in X^{n}$ we set

$$\begin{array}{cc} B(x^{n}) & =\left\{\;\left({\overset{ˇ}{x}}^{n}\right):\;H\left({\overset{ˇ}{x}}^{n}\right)\leq H\left(x^{n}\right)\;,\;P_{{\overset{ˇ}{x}}^{n}}=P_{x^{n}}\;\right\}, \end{array}$$

Using parts (a) and (b) of Lemma 4, we have following inequalities:

(A4) $$\begin{array}{cc} \left|B(x^{n})\right| & \leq {\left(n+1\right)}^{\left|X\right|}e^{nH(x^{n})}, \end{array}$$

On an upper bound of $E[\Xi_{x^{n}}\left(\phi^{\left(n\right)},\psi^{\left(n\right)}\right)]$, we have the following chain of inequalities:

E[Ξxn(ϕ(n),ψ(n))]≤∑xˇn∈B(xn),xˇn≠xnPrϕ(n)(xˇn)=ϕ(n)(xn)≤(a)∑xˇn∈B(xn)1M=|B(xn)|M≤(b)e(n+1)|X|e−n[R−H(xn)].

Step (a) follows from Lemma 7 part (a) and independent random constructions of linear encoders $\phi_{1}^{\left(n\right)}$ and $\phi_{e}^{\left(n\right)}$. Step (b) follows from (A4) and $M\geq e^{nR-1},i=1,2$. On the other hand we have the obvious bound $E[\Xi_{x^{n}}\left(\phi^{\left(n\right)},\psi^{\left(n\right)}\right)]\leq 1$. Hence we have

$$\begin{array}{c} E\left[\Xi_{x^{n}}\left(\phi^{\left(n\right)},\psi^{\left(n\right)}\right)\right]\leq e{\left(n+1\right)}^{\left|X\right|}\left\{e^{-n{\left[R-H\left(x^{n}\right)\right]}^{+}}\right\}. \end{array}$$

Hence we have

$$\begin{array}{cc} E\left[\Xi_{{\bar{X}}_{1}{\bar{X}}_{2}}\left(\phi^{\left(n\right)},\psi^{\left(n\right)}\right)\right]=E\left[\frac{1}{|T_{\bar{X}}^{n}|}\sum_{x^{n}\in T_{\bar{X}}^{n}}\Xi_{x^{n}}\left(\phi^{\left(n\right)},\psi^{\left(n\right)}\right)\right] & =\frac{1}{|T_{\bar{X}}^{n}|}\sum_{x^{n}\in T_{\bar{X}}^{n}}E\left[\Xi_{x^{n}}\left(\phi^{\left(n\right)},\psi^{\left(n\right)}\right)\right]\\ & \leq e{\left(n+1\right)}^{\left|X\right|}\left\{e^{-n{\left[R-H\left(\bar{X}\right)\right]}^{+}}\right\}, \end{array}$$

completing the proof. □

## Appendix D. Proof of Lemma 9

In this appendix, we prove Lemma 9. This lemma immediately follows from the following lemma:

**Lemma** **A1.**
*For any* n,m *satisfying* (m/n)log|X| ≤R *, we have*

(A5) $$\begin{array}{c} E\left[D\;\;\left.\left.\left.\left(p_{{\tilde{K}}^{m}|M_{A}^{\left(n\right)}}\right|\right|p_{V^{m}}\right|p_{M_{A}^{\left(n\right)}}\right)\right]\\ \leq \sum_{\left(a,k^{n}\right)\in M_{A}^{\left(n\right)}\times X^{n}}p_{M_{A}^{\left(n\right)}K^{n}}\left(a,k^{n}\right)\log\left[1+\left(\right|X^{m}\left|-1\right)p_{K^{n}|M_{A}^{\left(n\right)}}\left(k^{n}|a\right)\right]. \end{array}$$

In fact, from $|X^{m}|\leq e^{nR}$ and (A5) in Lemma A1, we have the bound (28) in Lemma 9. Thus, we prove Lemma A1 instead of proving Lemma 9.

In the following arguments, we use the following simplified notations:

$$\begin{array}{cc} k^{n},K^{n}\in X^{n} & ⟹k,K\in K,\\ {\tilde{k}}^{m},{\tilde{K}}^{m}\in X^{m} & ⟹l,L\in L,\\ \varphi^{\left(n\right)}:X^{n}\to X^{m} & ⟹\varphi :K\to L, \end{array}$$

$$\begin{array}{cc} \varphi^{\left(n\right)}\left(k^{n}\right)=k^{n}A+b^{m} & ⟹\varphi (k)=kA+b,\\ V^{m}\in X^{m} & ⟹V\in L,\\ M_{A}^{\left(n\right)}\in M_{A}^{\left(n\right)} & ⟹M\in M. \end{array}$$

We define

$$\chi_{\varphi (k),l}=\left\{\begin{array}{c} 1,\;\mathrm{if}\;\varphi (k)=l,\\ 0,\;\mathrm{if}\;\varphi (k)\neq l. \end{array}\right.$$

Then, the conditional distribution of the random variable $L=L_{\varphi }$ for given M=a∈M is

$$\begin{array}{c} p_{L|M}\left(l|a\right)=\sum_{k\in K}p_{K|M}\left(k|a\right)\chi_{\varphi (k),l}\;\mathrm{for}\;l\in L. \end{array}$$

Define

$$\begin{array}{c} \Upsilon_{\varphi (k),l}:=\chi_{\varphi (k),l}\log\left[\begin{array}{c} \;\\ \; \end{array}\right.\;\;\left|L\right|\left\{\begin{array}{c} \;\\ \; \end{array}\right.\;\;\sum_{k^{\prime }\in K}p_{K|M}\left(k^{\prime }|a\right)\chi_{\varphi (k^{\prime }),l}\left.\begin{array}{c} \;\\ \; \end{array}\;\;\right\}\left.\begin{array}{c} \;\\ \; \end{array}\;\;\right]. \end{array}$$

Then the conditional divergence between $p_{L|M}$ and $p_{V}$ for given *M* is given by

(A6) $$\begin{array}{c} \left.\left.\left.D\left(p_{L|M}\right|\right|p_{V}\right|p_{M}\right)=\sum_{(a,k)\in M\times K}\sum_{l\in L}p_{MK}\left(a,k\right)\Upsilon_{\varphi (k),l}. \end{array}$$

The quantity $\Upsilon_{\varphi (k),l}$ has the following form:

(A7) $$\begin{array}{c} \Upsilon_{\varphi (k),l}=\chi_{\varphi (k),l}\log\{\left|L\right|(p_{K|M}\left(k|a\right)\chi_{\varphi (k),l}\left.\left.+\sum_{k^{\prime }\in {\left\{k\right\}}^{c}}p_{K|M}\left(k^{\prime }|a\right)\chi_{\varphi (k^{\prime }),l}\right)\right\}. \end{array}$$

The above form is useful for computing $E[\Upsilon_{\varphi (k),l}]$.

**Proof** **of** **Lemma** **A1:** Taking the expectation of both sides of (A7) with respect to the random choice of the entry of the matrix *A* and the vector *b* representing the affine encoder *φ*, we have

(A8) $$\begin{array}{c} \left.\left.\left.E\left[D\left(p_{L|M}\right|\right|p_{V}\right|p_{M}\right)\right]=\sum_{(a,k)\in M\times K}\sum_{l\in L}p_{MK}\left(a,k\right)E\left[\Upsilon_{\varphi (k),l}\right]. \end{array}$$

To compute the expectation $E\left[\Upsilon_{\varphi (k),l}\right]$, we introduce an expectation operator useful for the computation. Let $E_{\varphi \left(k\right)=l_{k}}\left[\cdot \right]$ be an expectation operator based on the conditional probability measures $\mathrm{Pr}(\cdot |\varphi \left(k\right)=l_{k})$. Using this expectation operator, the quantity $E\left[\Upsilon_{\varphi (k),l}\right]$ can be written as

(A9) $$\begin{array}{c} E\left[\Upsilon_{\varphi (k),l}\right]=\sum_{l_{k}\in L}\mathrm{Pr}\left(\varphi \left(k\right)=l_{k}\right)E_{\varphi \left(k\right)=l_{k}}\left[\Upsilon_{l_{k},l}\right]. \end{array}$$

Note that

(A10) $$\Upsilon_{l_{k},l}=\left\{\begin{array}{cc} 1, & \mathrm{if}\;l_{k}=l,\\ 0, & \mathrm{otherwise}. \end{array}\right.$$

From (A9) and (A10), we have

(A11) $$\begin{array}{c} E\left[\Upsilon_{\varphi (k),l}\right]=\mathrm{Pr}\left(\varphi (k)=l\right)E_{\varphi (k)=l}\left[\Upsilon_{l,l}\right]=\frac{1}{\left|L\right|}E_{\varphi (k)=l}\left[\Upsilon_{l,l}\right]. \end{array}$$

Using (A7), the expectation $E_{\varphi (k)=l}\left[\Upsilon_{l,l}\right]$ can be written as

(A12) $$\begin{array}{c} E_{\varphi (k)=l}\left[\Upsilon_{l,l}\right]=E_{\varphi (k)=l}[\log\{\left|L\right|(p_{K|M}\left(k|a\right)\left.\left.\left.+\sum_{k^{\prime }\in {\left\{k\right\}}^{c}}p_{K|M}\left(k^{\prime }|a\right)\chi_{\varphi (k^{\prime }),l}\right)\right\}\right]. \end{array}$$

Applying Jensen’s inequality to the right member of (A12), we obtain the following upper bound of $E_{\varphi (k)=l}\left[\Upsilon_{l,l}\right]$:

(A13) $$\begin{array}{c} E_{\varphi (k)=l}\left[\Upsilon_{l,l}\right]\leq \log\{\left|L\right|(p_{K|M}\left(k|a\right)\left.\left.+\sum_{k^{\prime }\in {\left\{k\right\}}^{c}}p_{K|M}\left(k^{\prime }|a\right)E_{\varphi (k)=l}\left[\chi_{\varphi (k^{\prime }),l}\right]\right)\right\}\\ \overset{\left(a\right)}{=}\log\left\{\left|L\right|\left(p_{K|M}\left(k|a\right)+\sum_{k^{\prime }\in {\left\{k\right\}}^{c}}p_{K|M}\left(k^{\prime }|a\right)\frac{1}{\left|L\right|}\right)\right\}=\log\left\{1+\left(|L|-1\right)p_{K|M}\left(k|a\right)\right\}. \end{array}$$

Step (a) follows from that by Lemma 7 parts (b) and (c),

$$\begin{array}{cc} E_{\varphi (k)=l}\left[\chi_{\varphi (k^{\prime }),l}\right] & =\mathrm{Pr}\left(\varphi \left(k^{\prime }\right)=l|\varphi \left(k\right)=l\right)=\frac{1}{\left|L\right|}. \end{array}$$

From (A8), (A11), and (A13), we have the bound (A5) in Lemma A1. □

## Appendix E. Proof of Lemma 12

To prove Lemma 12, we prepare a lemma. For simplicity of notation, set $|M_{A}^{\left(n\right)}|=M_{A}$. Define

$$B_{n}:=\left\{\left(a,z^{n},k^{n}\right):\frac{1}{n}\log\frac{p_{M_{A}^{\left(n\right)}Z^{n}K^{n}}\left(a,z^{n},k^{n}\right)}{{\hat{q}}_{M_{A}^{\left(n\right)}Z^{n}K^{n}}\left(a,z^{n},k^{n}\right)}\geq -\eta \right\}.$$

Furthermore, define

$$\begin{array}{c} {\tilde{C}}_{n}:=\left\{z^{n}:\frac{1}{n}\log\frac{p_{Z^{n}}\left(z^{n}\right)}{q_{Z^{n}}\left(z^{n}\right)}\geq -\eta \right\},\\ C_{n}:={\tilde{C}}_{n}\times M_{A}^{\left(n\right)}\times X^{n},C_{n}^{c}:={\tilde{C}}_{n}^{c}\times M_{A}^{\left(n\right)}\times X^{n},\\ {\tilde{D}}_{n}:=\{\left(a,z^{n}\right):\begin{array}{c} a=\varphi_{A}^{\left(n\right)}\left(z^{n}\right),p_{Z^{n}|M_{A}^{\left(n\right)}}\left(z^{n}|a\right)\leq M_{A}e^{n\eta }p_{Z^{n}}\left(z^{n}\right)\}, \end{array}\\ D_{n}:={\tilde{D}}_{n}\times X^{n},D_{n}^{c}:={\tilde{D}}_{n}^{c}\times X^{n},\\ E_{n}:=\{\left(a,z^{n},k^{n}\right):\begin{array}{c} a=\varphi_{A}^{\left(n\right)}\left(z^{n}\right),p_{K^{n}|M_{A}^{\left(n\right)}}\left(k^{n}|a\right)\geq e^{-n(R+\eta )}\}. \end{array} \end{array}$$

Then we have the following lemma.

**Lemma** **A2.**

$$\begin{array}{cc} p_{M_{A}^{\left(n\right)}Z^{n}K^{n}}\left(B_{n}^{c}\right) & \leq e^{-n\eta },\\ p_{M_{A}^{\left(n\right)}Z^{n}K^{n}}\left(C_{n}^{c}\right) & \leq e^{-n\eta },\\ p_{M_{A}^{\left(n\right)}Z^{n}K^{n}}\left(D_{n}^{c}\right) & \leq e^{-n\eta }. \end{array}$$

**Proof.** We first prove the first inequality.

$$\begin{array}{cc} p_{M_{A}^{\left(n\right)}Z^{n}K^{n}}\left(B_{n}^{c}\right) & =\sum_{\left(a,z^{n},k^{n}\right)\in B_{n}^{c}}p_{M_{A}^{\left(n\right)}Z^{n}K^{n}}\left(a,z^{n},k^{n}\right)\\ & \overset{\left(a\right)}{\leq }\sum_{\left(a,z^{n},k^{n}\right)\in B_{n}^{c}}e^{-n\eta }{\hat{q}}_{M_{A}^{\left(n\right)}Z^{n}K^{n}}\left(a,z^{n},k^{n}\right)\\ & =e^{-n\eta }q_{M_{A}^{\left(n\right)}Z^{n}K^{n}}\left(B_{n}^{c}\right)\leq e^{-n\eta }. \end{array}$$

Step (a) follows from the definition of $B_{n}$. For the second inequality we have

$$\begin{array}{c} p_{M_{A}^{\left(n\right)}Z^{n}K^{n}}\left(C_{n}^{c}\right)=p_{Z^{n}}\left({\tilde{C}}_{n}^{c}\right)=\sum_{x^{n}\in {\tilde{C}}_{n}^{c}}p_{Z_{n}}\left(z^{n}\right)\\ \overset{\left(a\right)}{\leq }\sum_{x^{n}\in {\tilde{C}}_{n}^{c}}e^{-n\eta }q_{Z^{n}}\left(z^{n}\right)=e^{-n\eta }q_{Z^{n}}\left({\tilde{C}}_{n}^{c}\right)\leq e^{-n\eta }. \end{array}$$

Step (a) follows from the definition of $C_{n}$. We finally prove the third inequality.

pMA(n)ZnKn(Dnc)=pMA(n)Zn(D˜nc)=∑a∈MA(n)∑zn:φA(n)(zn)=apZn(zn)≤(e−nη/MA)×pZn|MA(n)(zn|a)pZn(zn)≤e−nηMA∑a∈MA(n)∑zn:φA(n)(zn)=apZn(zn)≤(e−nη/MA)×pZn|MA(n)(zn|a)pZn|MA(n)(zn|a)≤e−nηMA|MA(n)|=e−nη.

This completes the proof of Lemma A2. □

**Proof** **of** **Lemma** **12:** By definition, we have

$$\begin{array}{c} p_{M_{A}^{\left(n\right)}Z^{n}K^{n}}\left(B_{n}\cap C_{n}\cap D_{n}\cap E_{n}\right)\\ =p_{M_{A}^{\left(n\right)}Z^{n}K^{n}}\left\{\frac{1}{n}\log\frac{p_{M_{A}^{\left(n\right)}Z^{n}K^{n}}\left(M_{A}^{\left(n\right)},Z^{n},K^{n}\right)}{{\hat{q}}_{M_{A}^{\left(n\right)}Z^{n}K^{n}}\left(M_{A}^{\left(n\right)},Z^{n},K^{n}\right)}\geq -\eta ,\right.\\ \;\;\;\;0\geq \frac{1}{n}\log\frac{q_{Z^{n}}\left(Z^{n}\right)}{p_{Z^{n}}\left(Z^{n}\right)}-\eta ,\\ \;\;\;\frac{1}{n}\log M_{A}\geq \frac{1}{n}\log\frac{p_{Z^{n}|M_{A}^{\left(n\right)}}\left(Z^{n}|M_{A}^{\left(n\right)}\right)}{p_{Z^{n}}\left(Z^{n}\right)}-\eta ,\\ \;\;\;R\geq \left.\frac{1}{n}\log\frac{1}{p_{K^{n}|M_{A}^{\left(n\right)}}\left(K^{n}|M_{A}^{\left(n\right)}\right)}-\eta \right\}. \end{array}$$

Then for any $\varphi_{A}^{\left(n\right)}$ satisfying $\left(1/n\right)\log\left|\right|\varphi_{A}^{\left(n\right)}\left|\right|\leq R_{A},$ we have

$$\begin{array}{c} p_{M_{A}^{\left(n\right)}Z^{n}K^{n}}\left(B_{n}\cap C_{n}\cap D_{n}\cap E_{n}\right)\\ \leq p_{M_{A}^{\left(n\right)}Z^{n}K^{n}}\left\{\frac{1}{n}\log\frac{p_{M_{A}^{\left(n\right)}Z^{n}K^{n}}\left(M_{A}^{\left(n\right)},Z^{n},K^{n}\right)}{{\hat{q}}_{M_{A}^{\left(n\right)}Z^{n}K^{n}}\left(M_{A}^{\left(n\right)},Z^{n},K^{n}\right)}\geq -\eta ,\right.\\ \;\;0\geq \frac{1}{n}\log\frac{q_{Z^{n}}\left(Z^{n}\right)}{p_{Z^{n}}\left(Z^{n}\right)}-\eta ,\\ \;R_{A}\geq \frac{1}{n}\log\frac{p_{Z^{n}|M_{A}^{\left(n\right)}}\left(Z^{n}|M_{A}^{\left(n\right)}\right)}{p_{Z^{n}}\left(Z^{n}\right)}-\eta ,\\ \;R\geq \left.\frac{1}{n}\log\frac{1}{p_{K^{n}|M_{A}^{\left(n\right)}}\left(K^{n}|M_{A}^{\left(n\right)}\right)}-\eta \right\}. \end{array}$$

Hence, it suffices to show

$$\begin{array}{c} {℘}_{\eta }^{\left(n\right)}\leq p_{M_{A}^{\left(n\right)}Z^{n}K^{n}}\left(B_{n}\cap C_{n}\cap D_{n}\cap E_{n}\right)+3e^{-n\eta } \end{array}$$

to prove Lemma 12. We have the following chain of inequalities:

$$\begin{array}{cc} ℘ & \overset{\left(a\right)}{=}p_{M_{A}^{\left(n\right)}Z^{n}K^{n}}\left(E_{n}\right)\\ & =p_{M_{A}^{\left(n\right)}Z^{n}K^{n}}\left(B_{n}\cap C_{n}\cap D_{n}\cap E_{n}\right)+p_{M_{A}^{\left(n\right)}Z^{n}K^{n}}\left({\left[B_{n}\cap C_{n}\cap D_{n}\right]}^{c}\cap E_{n}\right)\\ & \leq p_{M_{A}^{\left(n\right)}Z^{n}K^{n}}\left(B_{n}\cap C_{n}\cap D_{n}\cap E_{n}\right)+p_{M_{A}^{\left(n\right)}Z^{n}K^{n}}\left(B_{n}^{c}\right)+p_{M_{A}^{\left(n\right)}Z^{n}K^{n}}\left(C_{n}^{c}\right)+p_{M_{A}^{\left(n\right)}Z^{n}K^{n}}\left(D_{n}^{c}\right)\\ & \overset{\left(b\right)}{\leq }p_{M_{A}^{\left(n\right)}Z^{n}K^{n}}\left(B_{n}\cap C_{n}\cap D_{n}\cap E_{n}\right)+3e^{-n\eta }=\tilde{℘}. \end{array}$$

Step (a) follows from the defintion of ℘. Step (b) follows from Lemma A2. ☐
